# FedCARE: Fuzzy-Supervised Federated Inference with Confidence Gating for Resilient IIoT Sensor Networks

**DOI:** 10.3390/s26123904

**Published:** 2026-06-19

**Authors:** Basma Mostafa, Hanan Haj Ahmad, Yazan Rabaiah, Marwa Elseddik

**Affiliations:** 1 Operations Research Department, Faculty of Computers and Artificial Intelligence, Cairo University, Giza 12613, Egypt; basma.mostafa@cu.edu.eg; 2 Faculty of Artificial Intelligence & Informatics, Horus University, New Damietta 34518, Egypt; 3 Department of Mathematics and Statistics, College of Science, King Faisal University, Hofuf 31982, Al-Ahsa, Saudi Arabia; 4 Department of Electrical Engineering, College of Engineering, King Faisal University, Hofuf 31982, Al-Ahsa, Saudi Arabia; 225072774@student.kfu.edu.sa; 5 Department of Robotics and Intelligent Machines, Faculty of Artificial Intelligence, Kafrelsheikh University, Kafr El-Sheikh 33516, Egypt; marwaelsadeek@ai.kfs.edu.eg

**Keywords:** federated learning, sensor networks, IIoT security, fuzzy inference, confidence-gated fusion, edge intelligence, dropout-aware aggregation, criticality-aware routing, Internet of Things, cyber-physical systems

## Abstract

Safety-critical Industrial Internet of Things (IIoT) sensor networks deployed in disaster scenarios require intelligent routing mechanisms that prioritize mission-critical packets without relying on centralized coordination. Federated learning on resource-constrained edge nodes presents three primary challenges: the absence of an interpretable supervisory signal, the inability to act conservatively based on per-inference confidence, and vulnerability to partial node availability. The proposed FedCARE framework addresses these issues by employing a Mamdani Fuzzy Inference System to generate traceable criticality labels from multi-modal sensor telemetry, a dropout-aware aggregation protocol that normalizes over only reachable nodes, and a confidence-gated resolver that defers to symbolic fuzzy classification when model confidence is insufficient, otherwise applying an auditable maximization rule to prevent under-prioritization of safety-critical data. Evaluation on 50-, 100-, and 200-node Watts–Strogatz topologies under fault rates up to 50%, using the Edge-IIoTset and WUSTL-IIoT-2021 benchmarks, demonstrates 99.00% critical recall and up to 1.8× higher overall-packet delivery compared to RPL-RP under severe fault conditions. Routing improvements are primarily attributed to fuzzy criticality labeling and multi-path replication. These findings indicate that fuzzy-supervised federated inference offers a practical and interpretable solution for safety-critical IIoT routing, with an observed energy overhead of 7.8% per delivered packet.

## 1. Introduction

Machine learning systems deployed in safety-critical Industrial Internet of Things (IIoT) environments operate under constraints that fundamentally differ from those assumed in conventional machine learning system design. Edge nodes are subject to memory and energy constraints, and network connectivity is often intermittent. Data distributions may shift unpredictably under fault conditions. The cost of misclassification is asymmetric. Failure to identify a critical threat presents significantly greater risk than a false positive. Natural and human-made disasters such as earthquakes, floods, wildfires, industrial accidents, and coordinated cyber-attacks intensify these constraints by causing widespread node failures, with up to 50% sudden loss [1,2], intermittent connectivity of fog gateways, extreme link congestion, and cascading sensor outages. Empirical reviews of IoT deployments in disaster scenarios confirm that such failure rates render conventional sensor-node monitoring and classification pipelines largely ineffective [1,3,4].

In this study, federated learning (FL) denotes a distributed machine learning paradigm in which multiple IIoT edge nodes collaboratively train a shared model without transmitting raw sensor or traffic data to a central server. Only local model updates are exchanged with a fog-level aggregator, thereby preserving data locality and reducing the need for centralized data collection. The term IIoT threat detection refers to the identification of abnormal cyber-physical conditions in industrial sensor networks, including malicious traffic patterns such as denial-of-service or spoofing attacks, as well as safety-critical physical events reflected in sensor readings such as flame, temperature, vibration, latency, residual energy, and hop-distance measurements. Within FedCARE, threat detection extends beyond conventional intrusion classification and is integrated with criticality-aware routing, enabling detected or inferred high-risk events to trigger more reliable forwarding actions.

These conditions expose three specific and largely unaddressed gaps in the machine learning literature as it applies to constrained cyber-physical systems.

The first research gap pertains to model supervision under resource constraints. Federated Learning (FL) has emerged as the primary approach for collaborative model training across distributed IIoT nodes without sharing raw telemetry [5,6]. However, FL models for IIoT threat detection rely on either manually annotated or synthetically generated labels, which are not reliably available at the network edge during disaster scenarios. Currently, no framework provides an interpretable, domain-consistent, and automatically generated labeling mechanism that encodes physical network semantics directly into the federated model’s supervision signal [7,8].

The second gap concerns confidence quantification and conservative inference fusion. Existing FL-based classification systems for IIoT provide only a predicted class, without per-inference confidence estimates or a principled mechanism for conservative action when predictions are unreliable [9,10]. This is a structural deficiency in safety-critical environments. A model experiencing cold start, distributional shift, or client dropout has no mechanism to signal its own unreliability, and the system lacks a fallback strategy. As a result, uncertain predictions are treated with the same authority as confident ones, which is not justified when the downstream cost of under-prioritizing a critical threat is severe [11,12].

The third research gap addresses the resilience of federated aggregation under limited client availability. Standard FedAvg and its variants require a large, stable pool of clients in each aggregation round [13,14]. Adaptive methods such as FedProx and SCAFFOLD employ proximal regularization or variance reduction to improve robustness. However, these approaches do not account for the sudden, unpredictable loss of IIoT clients and fog gateway connectivity that can occur during disasters. Availability is neither stable nor predictable. Standard aggregation protocols do not normalize over the active client set. As a result, node failures introduce systematic bias into the global model, and there is no graceful degradation mechanism when connectivity to the fog aggregator is lost entirely [15].

To address the identified research gaps, this study introduces FedCARE (Criticality-Aware Routing Engine), a federated edge-intelligence framework for Industrial Internet of Things (IIoT) sensor networks. Each contribution is substantiated by multi-modal sensor telemetry, including latency measurements, residual energy readings, and hop-distance counts. The framework delivers three coordinated machine learning contributions, each specifically aligned with a corresponding research gap. Data are collected from individual sensor nodes, directly connecting the federated learning (FL) pipeline to physical network observables. This approach grounds the system in sensor data, ensuring that model training and inference are based on real-world measurements. This integration enhances the framework’s relevance in the sensor network domain.

Gap 1: Fuzzy supervision is addressed by employing a Mamdani Fuzzy Inference System [16,17] as an interpretable offline supervisor. This system maps heterogeneous physical path metrics to expert-informed criticality labels through a transparent linguistic rule base. The resulting knowledge-extraction pipeline generates a domain-consistent, automatically produced training signal without manual annotation. All outputs are fully traceable to identifiable rule firings [18,19].

Gap 2: Confidence-gated resolution is achieved using a criticality resolver that evaluates per-inference confidence against a configurable threshold. If confidence is insufficient, the resolver defers to symbolic fuzzy classification, suppressing unstable predictions during cold starts or distributional shifts. If confidence is sufficient, an auditable maximization rule, $c^{★}$ = $\max(c_{f},c_{m})$, is applied to ensure that neither inference layer can override a higher severity classification produced by the other [11].

In the routing experiments reported here, the FL model operates in conservative cold-start deferral mode throughout. Its independent contribution to classification accuracy is validated on the Edge-IIoTset benchmark (99.00% critical recall). The resolved criticality class is then mapped deterministically to a forwarding policy, closing the loop between federated inference and physical network actuation without reliance on any opaque fusion mechanism [20,21].

Gap 3: Dropout-Aware Aggregation. A dropout-aware federated aggregation protocol constructs an active-client set at each global round, normalizes weights only over reachable participants, and gracefully degrades to the last valid model when no clients are available. Round-level convergence metrics are logged continuously [22,23].

The main contributions of this paper are as follows.

- Fuzzy-Supervised Label Generation for Federated Learning. A Mamdani Fuzzy Inference System generates expert-informed criticality labels from multi-modal sensor telemetry (latency, residual energy, hop distance), providing a traceable, domain-consistent supervision signal for a lightweight FL model and eliminating the dependence on manual annotation or centralized labeling for safety-critical IIoT inference [16,17,18].
- Dropout-Aware Federated Aggregation. A federated synchronization protocol normalizes aggregation exclusively over the active-client set at each round, prevents absent nodes from biasing the global model, and provides graceful degradation and continuous convergence monitoring under the partial connectivity characteristic of disaster scenarios [6,22].
- Confidence-Gated Criticality Resolution. A principled inference fusion mechanism suppresses unstable federated predictions during cold-start and distributional shift by gating on per-inference confidence, and applies an auditable maximization rule when confidence is sufficient, guaranteeing conservative fail-safe behavior without any opaque fusion. The FL component’s routing contribution is validated separately on Edge-IIoTset, where full model convergence yields 99.00% critical recall [9].
- Criticality-Conditioned Network Actuation. The resolved criticality class is mapped directly to deterministic IPv6 forwarding actions—single-path routing, bounded retransmissions, or *k*-path spatial replication—using additive RPL-compatible metrics with O(1) per-hop computation, demonstrating that ML-driven inference can close the loop to physical actuation within the strict computational budget of constrained IIoT motes [19,20,21].
- Statistically Grounded Multi-Scale Evaluation. FedCARE is validated using the Edge-IIoTset [24] and WUSTL-IIoT-2021 [25] datasets—the former for classification accuracy on cyber-attack signatures, the latter for intrusion pattern diversity—in a hybrid Contiki-Cooja simulation across 50-, 100-, and 200-node topologies under fault rates up to 50%, with multi-seed statistical analysis and Holm–Bonferroni correction. FedCARE achieves 99.00% critical recall and up to 1.8× higher high-criticality delivery than RPL-RP, the strongest routing baseline, under severe 50% node-failure conditions at the 200-node scale.
- Fairness–Reliability–Energy Characterization. The operational trade-off introduced by criticality prioritization is quantified across network scales and fault rates. Under 50% fault injection, FedCARE’s 1.8× reliability gain is achieved at approximately 7.8% higher energy per packet and a 20 pp delivery gap between critical and low-priority flows, bounding the deployment regimes where prioritization is cost-justified.
- Practical Applicability of FedCARE in IIoT Scenarios. FedCARE is designed for safety-critical IIoT environments that require local sensor data processing and reliable delivery of critical alerts, even when nodes fail or connectivity is unstable. It is suitable for smart factories, smart grids, disaster-response networks, oil and gas facilities, chemical plants, and mining sites to detect cyber-physical risks such as fire, overheating, vibration anomalies, spoofed commands, equipment faults, and network attacks. Unlike solutions that only classify threats, FedCARE links the detected criticality level directly to routing actions, including single-path forwarding, bounded retransmissions, or multi-path replication. This approach ensures reliable delivery of high-priority alerts, interpretability, and resilient operation under failures, which are often more important than energy efficiency alone.

The remainder of this paper is organized as follows. Section 2 reviews related work on federated learning for IIoT security, fuzzy inference for knowledge extraction, and reliability-aware routing—the three pillars that FedCARE integrates. Section 3 formalizes the FedCARE system model and Section 4 presents the four-component algorithmic workflow. Section 5 details the evaluation methodology, datasets, and statistical protocol. Section 6 presents and analyzes experimental results across all scales and fault regimes. Section 7 states the scope boundaries of the reported results. Section 8 concludes and identifies directions for future research.

## 2. Literature Review

Research relevant to FedCARE spans three principal axes in the machine learning literature: interpretable, knowledge-driven inference for cyber-physical systems; federated learning for distributed IIoT security; and learning-augmented reliability-aware routing. Across these axes, a recurring pattern emerges: systems that generate interpretable inferences typically do not actuate the network, while those that actuate the network often lack transparent reasoning. The primary gap is the absence of a closed-loop machine learning architecture that integrates interpretable inference, federated learning, and physical actuation within a unified pipeline. Table 1 situates FedCARE within this research context. Table 2 quantifies the specific research gaps and their operational impacts.

### 2.1. Interpretable Inference and Knowledge Extraction for Cyber-Physical Systems

Extracting actionable knowledge from heterogeneous physical sensor streams remains a central challenge in IIoT machine learning. Existing IIoT frameworks generally encode task criticality—defined as the consequence of task failure on mission outcomes—using rigid, design-time mechanisms such as static priorities and deadlines. Mixed-Criticality Systems [26]. Because these assignments are fixed at design time, they cannot accommodate the dynamic evolution of physical hazards or cascading actuator failures during emergencies. This approach is incompatible with the core ML requirement for adaptive, data-driven reasoning under distributional shift.

Fuzzy Inference Systems (FIS), and the Mamdani model in particular [16], have been adopted as interpretable alternatives that encode domain expertise as a transparent linguistic rule base and produce outputs traceable to identifiable rule firings [17,18]. Unlike black-box classifiers, a Mamdani FIS provides a quantifiable explanation mechanism: every output is the result of named linguistic rules applied to named input variables, making it directly suitable as a knowledge-extraction and label-generation layer. However, most existing fuzzy approaches function as standalone decision-makers rather than as supervisory components that generate training signals for the downstream learned models [18,19]. The potential of fuzzy inference as an interpretable offline supervisor for federated learning remains unexplored.

Explainable AI methods for cyber-physical systems have similarly been limited to passive diagnosis. Frameworks based on deep learning augmented with SHAP or LIME provide interpretable attack attribution and process anomaly explanation [11,12], and analytical models map dependencies between sensor inputs and control actuators. These systems improve transparency but lack a mechanism to translate inferences into adaptive control actions, leaving the reasoning-to-action chain incomplete. FedCARE addresses this by using the Mamdani FIS not as a final decision-maker but as an interpretable knowledge extractor whose outputs supervise a federated model and govern a downstream actuation policy.

### 2.2. Federated Learning for IIoT Security

Federated learning has emerged as the principal mechanism for collaborative model training on distributed IIoT sensor nodes without sharing raw telemetry, making it well suited for privacy-sensitive industrial deployments [5,6]. FL-based Intrusion Detection Systems (FL-IDS) apply this principle to network security, enabling distributed anomaly detection while preserving data locality [8,41]. Recent work has demonstrated that FL can substantially reduce backhaul communication overhead while maintaining detection accuracy on constrained sensor nodes [22,23], and federated risk assessment frameworks employing XGBoost and SVM models provide distributed risk scoring with interpretable outputs [7,9].

An emerging strand augments FL-IDS with explainability methods. Explainable FL-IDS architectures incorporating SHAP and LIME have been proposed for IIoT, IoT, and smart building applications [10], improving transparency and operator trust. Adaptive aggregation schemes—such as Shapley value-based weighting across heterogeneous IIoT clients [27]—improve robustness to non-IID data, but focus exclusively on improving model accuracy rather than coupling inference outputs to network-layer decisions. These contributions are valuable but share a common structural limitation: FL outputs terminate at a classification or risk score. No existing FL-IDS framework couples its inference outputs to network-layer routing decisions or reliability controls, meaning that high detection accuracy does not guarantee delivery of the detected alert. This gap, which FedCARE explicitly closes by mapping federated criticality estimates to differentiated forwarding policies, is summarized in the Axis I and Axis II rows of Table 1.

Three additional ML-specific limitations distinguish the existing FL literature from FedCARE’s design. First, existing FL-IDS systems provide no per-inference confidence estimate, meaning that unstable predictions during model cold-start or distributional shift are acted upon with the same authority as confident ones [9,10]. Second, standard FedAvg and its variants normalize aggregation over all registered clients rather than only the active subset, introducing systematic bias when sensor nodes drop out [13,14]. Third, no existing framework provides an interpretable, automatically generated labeling mechanism for federated model supervision, forcing dependence on manual annotation or synthetic data [8]. FedCARE addresses all three through its confidence-gated resolver, dropout-aware aggregation protocol, and fuzzy-supervised label generation pipeline.

### 2.3. Learning-Augmented Reliability-Aware Routing

Routing protocols for Low-Power and Lossy Networks (LLNs) have evolved considerably beyond static metrics such as Expected Transmission Count (ETX) [20,21]. Recent ML-augmented RPL variants demonstrate that learned metrics substantially improve forwarding decisions under dynamic conditions. RPL+ applies a Random Forest to ETX, MAC-layer losses, and throughput for adaptive parent selection [29]; ML-RPL employs Gradient Boosted Decision Trees for predictive routing [30]; and RI-RPL integrates Q-learning for adaptive candidate selection [31]. Fuzzy logic has also been incorporated at the routing layer: the approach of Darabkh et al. [19] uses a cross-layer fuzzy system integrating hop count, energy, latency, and RSSI for parent selection. Energy-aware routing with node fault prediction for IoT sensor networks has further demonstrated that QoS-aware forwarding can adapt to node failures in real time [32].

Despite these advances, two deficiencies persist. First, all existing ML-routing frameworks treat packets as semantically homogeneous, optimizing network-layer metrics without awareness of the operational urgency of the underlying sensor payload—a limitation termed semantic blindness [26]. This contrasts with Mission-Critical IoT (MC-IoT) standards, where 3GPP and IETF define stringent reliability (≥99.999%) and latency (≤1 ms) targets for safety-critical traffic. Existing MC-IoT solutions address these targets at the PHY and MAC layers through ultra-reliable low-latency communication (URLLC) techniques, but they do not incorporate application-layer semantic awareness or federated threat inference, leaving them blind to coordinated cyber-physical attacks that manifest as anomalous payload patterns rather than link failures. Second, Deep Reinforcement Learning routing agents achieve adaptability at the cost of interpretability [33]: their forwarding decisions are not traceable to symbolic rules or quantified confidence measures, and their exploration–exploitation phases introduce unacceptable convergence latency under the rapid topology changes in disaster scenarios. Intrusion Prevention Systems (IPS) do address both detection and network-layer response, but they operate on centralized traffic mirrors and apply coarse-grained blocking rules, neither of which is feasible on the 8 KB RAM budget of constrained IIoT sensor motes or compatible with decentralized federated inference. FedCARE’s contribution is distinct: it couples per-packet criticality inference directly to the forwarding policy at the edge node, providing fine-grained priority differentiation rather than binary block-or-pass decisions, without any centralized vantage point. Disaster-aware networking protocols address physical infrastructure damage, but integrate no learned threat intelligence [1,37]. The axis III rows of Table 1 highlight these limitations relative to FedCARE.

### 2.4. Challenges and Research Gaps

The combination of federated learning operational assumptions and the constraints of post-disaster IIoT sensor environments creates compounding ML-specific challenges that no existing framework resolves. Industrial device heterogeneity leads to non-IID data streams that slow FL convergence and bias the global model [13]. Cryptographic and differential privacy mechanisms deployed to combat gradient leakage increase computational strain and communication latency on resource-constrained sensor nodes, creating network bottlenecks [14,42,43]. Frequent large model updates cause straggler effects that cascade into unacceptable latency spikes in post-disaster environments with severe multipath fading and infrastructure damage [15]. Scalability challenges include increased communication overhead as sensor networks grow, insufficient aggregation timing optimization for non-stationary environments, and the absence of standardized interoperability frameworks across heterogeneous industrial platforms [39,40]. As summarized in Table 2, the fundamental gap is the absence of architectures that simultaneously provide interpretable and automatically supervised FL inference, conservative confidence-aware fusion, and real-time disaster-resilient actuation.

### 2.5. Positioning of FedCARE

As Table 1 demonstrates, current approaches operate in isolation across three dimensions: fuzzy systems provide interpretability without learning; FL systems provide learned inference without network actuation; and routing protocols provide actuation without semantic awareness. FedCARE integrates all three into a unified closed-loop machine learning pipeline. Rather than appending post hoc explanation methods to a pre-trained model, FedCARE uses the Mamdani FIS [16] as an upstream knowledge extractor whose linguistically interpretable outputs directly supervise the federated model, ensuring that learned criticality classes inherit the semantic grounding of physical domain expertise. The confidence-gated resolver enables the fusion of symbolic and learned inference, with every decision point auditable. The dropout-aware aggregation protocol makes federated training robust to the partial connectivity of disaster topologies, normalizing exclusively over the active sensor node set rather than all registered clients [6,22]. The actuation layer maps the resolved criticality class to deterministic forwarding policies whose branching conditions are fully transparent [20,21]. By operationalizing ML inference outputs as control inputs for the network layer, FedCARE addresses semantic blindness and closes the reasoning-to-action chain that existing frameworks leave open [8,10].

Beyond the single-axis comparison, more rigorous evaluation of FedCARE’s contribution compares it to works that integrate two of its three pillars: learned inference, federated training, and routing actuation. Four such partial integrations represent the closest prior art. First, federated DRL routing combines federated learning with reinforcement-learned forwarding. Manogaran et al. [34] train an adaptive double deep Q-learning router (ADDQL, tuned by an improved hippopotamus optimizer) across WSN nodes under FL coordination, and Wang et al. [35] propose federated reinforcement learning for QoS- and privacy-aware routing in 5G-enabled IIoT. Both achieve decentralized adaptability, yet the learned policy is a black-box value function: forwarding decisions are not traceable to symbolic rules, and no per-inference confidence is quantified. Critically, packets are treated as semantically homogeneous, so routine telemetry readings and gas-leak alarms receive identical forwarding treatment.

Second, intelligent fault-tolerant routing addresses resilience. Kaur and Chanak [36] detect and recover node and link faults in WSN-assisted IIoT using hybrid reinforcement learning, which improves delivery under failures. However, their concept of criticality applies only to network elements, not to the inferred urgency of the payload, and no federated model is trained.

Third, secure and trustworthy FL hardens the learning loop itself. Issa et al. [28] combine digital twins and blockchain to purify poisoned updates in IoT federated learning, while fuzzy logic-based secure routing incorporates trust-aware metrics into hierarchical forwarding without federated training [44]. In both, the pipeline ends at model integrity or node selection and never conditions per-packet forwarding on inferred criticality.

Fourth, FL-optimization works treat learning efficiency as the end goal. Deep reinforcement learning is used to select devices and schedule rounds in digital-twin-empowered IIoT [13] or to offload computation in digital twin networks [43]. These contributions are orthogonal to FedCARE’s since the optimized model output is never linked to a network-layer control action.

This analysis, consistent with gaps identified in recent surveys of federated learning at the IIoT edge [22,45], shows that no existing system combines (i) interpretable fuzzy supervision that automatically generates criticality labels, (ii) federated training that is robust to client dropout, (iii) per-inference confidence gating between symbolic and learned paths, and (iv) criticality-conditioned routing actuation. The closest two-pillar systems forfeit exactly the properties that the ablation study isolates empirically: removing the fuzzy supervisory engine (Configuration B, Table 3) costs 6.2 percentage points of overall PDR at 200 nodes under 50% faults, and disabling the confidence gate removes the cold-start safety guarantee under which the learned path is deferred whenever $\kappa_{m}<\gamma$. FedCARE’s contribution is therefore not any individual component. Each is deliberately standard, but the closed Perception–Cognition–Actuation loop binds them, with every hand-off (label generation, aggregation, gating, forwarding) auditable and evaluated under identical fault-injection conditions (Section 6.3).

## 3. The FedCARE Architecture

**Figure 1:**
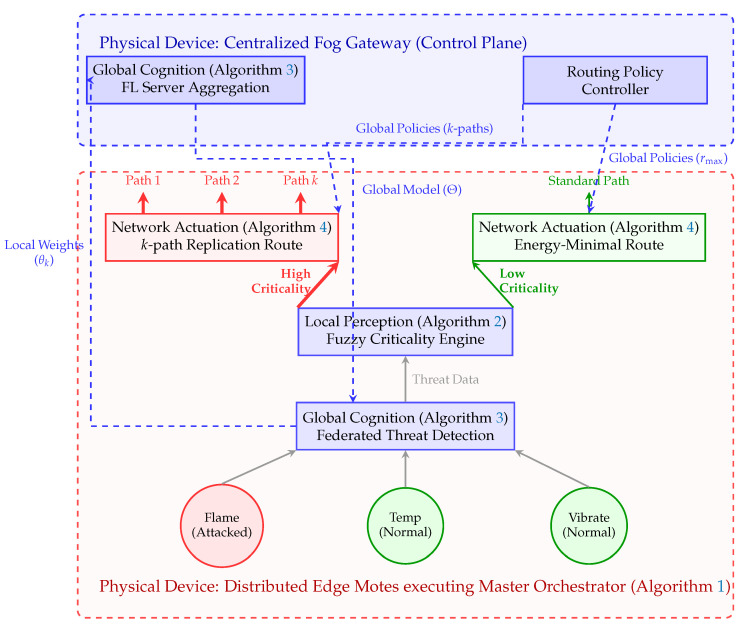
The FedCARE Perception–Cognition–Actuation (PCA) architecture. The Master Orchestrator (Algorithm 1) runs entirely within the physical boundary of the edge motes. Raw sensor data are evaluated sequentially by Local Perception (Algorithm 2) and Global Cognition (Algorithm 3) to execute a conservative confidence-gated threat override. The resolved criticality physically triggers Network Actuation (Algorithm 4), where severe threats spawn survival-oriented *k*-path routes and safe traffic defaults to energy-minimal routing.

FedCARE (Federated Criticality-Aware Routing Engine) is a cross-layer architecture that bridges the gap between decentralized edge intelligence and network-layer actuation in resource-constrained IIoT environments. FedCARE ensures safety-critical routing decisions execute with minimal latency while leveraging global threat insights by adopting a hierarchical edge–fog design that explicitly decouples the local Data Plane, executing on distributed Z1 edge motes, from the global Control Plane, managed centrally by a fog gateway. This separation follows the established pattern of hierarchical IoT architectures in which edge nodes perform low-latency local inference while the fog layer coordinates global policy [22]. As illustrated in Figure 1, the architecture comprises four functional layers: three operate entirely on the physical edge motes, and one is orchestrated globally from the fog gateway.

**Algorithm 1** Robust Edge Agent Orchestration **Require:** Stream S, local graph $G_{\mathrm{local}}$, current model Θ, config C **Ensure:** 
Stable criticality-aware forwarding under uncertainty  1:**while true do**  2:    p← ReadSensorStream(S)  3:    **if** p=⌀ **then**  4:        **continue**                                                                                                  ▹ Sensor-stall guard: skip invalid reads  5:    **end if**  6:    $E_{\mathrm{res}}\leftarrow$ GetResidualBattery()  7:    (λ,ε,δ)← ExtractPathMetrics(*p*, *G*_local_)  8:    **if** Invalid(λ,ε,δ) **then**  9:        (λ,ε,δ)← SafeDefaults()                                                                       ▹ Substitute pre-configured safe values10:    **end if**11:    $\left(\sigma_{f},c_{f}\right)\leftarrow$ FuzzyInference(*λ*, *ε*, *δ*)                                                                       ▹ Local Perception (Algorithm 2)12:    $\left(\sigma_{m},c_{m},\kappa_{m}\right)\leftarrow$ ModelPredict(F(p),Θ)                         ▹ Global Cognition: Predictive Inference (Algorithm 3)13:    **if** $\kappa_{m}<C.\gamma$ **then**14:        $c^{★}\leftarrow c_{f}$                                                                                           ▹ Confidence gate: defer to physical heuristic15:    **else**16:        $c^{★}\leftarrow \max\left(c_{f},c_{m}\right)$                                                                                                    ▹ Conservative threat override17:    **end if**18:    AdaptiveRouting$\left(p,dst,c^{★},G_{\mathrm{local}},C,E_{\mathrm{res}}\right)$                                                         ▹ Network Actuation (Algorithm 4)19:    **if** TimeToSync(C)** and not** IsTraining() **then**20:        **spawn async** Θ← FedLearningRound(*Θ*)                                ▹ Background FL sync; atomic model swap on completion21:    **end if**22:**end while**
**Algorithm 2** Local Perception via Fuzzy Risk Assessment **Require:** Path metrics (λ,ε,δ), membership functions M, rule base R, thresholds T **Ensure:** $\sigma_{f}\in \left[0,1\right]$, $c_{f}\in \left\{0,1,2\right\}$  1:$\left(\tilde{\lambda },\tilde{\varepsilon },\tilde{\delta }\right)\leftarrow$ Fuzzify(λ,ε,δ,M)                                                                    ▹ Step 1: Metric Fuzzification  2:$\sigma_{f}\leftarrow$ MamdaniInference$\left(\tilde{\lambda },\tilde{\varepsilon },\tilde{\delta },R\right)$           ▹ Step 2: Rule Evaluation and Centroid Defuzzification  3:**if** $\sigma_{f}\geq \tau_{\mathrm{high}}$** then**                                                                                       ▹ Step 3: Policy Discretization  4:    $c_{f}\leftarrow 2$                                                                                       ▹ High—triggers $k_{\mathrm{rep}}$-path replication  5:**else if** $\sigma_{f}\geq \tau_{\mathrm{med}}$ **then**  6:    $c_{f}\leftarrow 1$                                                                ▹ Medium—triggers bounded MAC retransmissions  7:**else**  8:    $c_{f}\leftarrow 0$                                                                            ▹ Low—energy-minimal single-path routing  9:**end if**10:**return**$\left(\sigma_{f},c_{f}\right)$

**Algorithm 3** Dropout-Aware Federated Synchronization
 **Require:** Previous global model $\Theta^{\left(r-1\right)}$, local datasets ${\left\{D_{k}\right\}}_{k=1}^{K}$, local epochs *E*, learning rate η **Ensure:** Updated global model $\Theta^{\left(r\right)}$
  1:

$K_{r}\leftarrow ⌀$

  2:**for each** client k∈{1,…,K} **in parallel do**  3:    **if** ClientAvailable(k) **then**  4:        $\theta_{k}^{\left(r\right)}\leftarrow$ ClientUpdate $\left(\Theta^{\left(r-1\right)},D_{k},E,\eta \right)$  5:        Upload $\left(\theta_{k}^{\left(r\right)},\left|D_{k}\right|\right)$ to aggregator  6:        $K_{r}\leftarrow K_{r}\cup \left\{k\right\}$  7:    **end if**  8:
**end for**
  9:**if** $|K_{r}|=0$ **then**10:    **return** $\Theta^{\left(r-1\right)}$               ▹ Graceful degradation: no available clients11:
**end if**
12:$\Theta^{\left(r\right)}\leftarrow \sum_{k\in K_{r}}\frac{|D_{k}|}{\sum_{j\in K_{r}}\left|D_{j}\right|}\theta_{k}^{\left(r\right)}$        ▹ Normalized aggregation over active clients only13:$\left(\ell_{r},{\mathrm{Acc}}_{r}\right)\leftarrow$ EvaluateValidation$\left(\Theta^{\left(r\right)}\right)$14:
LogRoundMetrics $\left(r,|K_{r}|,\ell_{r},{\mathrm{Acc}}_{r}\right)$

15:
**return** $\Theta^{\left(r\right)}$




**Algorithm 4** Reliability-Aware Adaptive Routing
 **Require:** Packet *p*, destination dst, criticality $c^{★}$, local graph $G_{\mathrm{local}}$, config C, residual energy $E_{\mathrm{res}}$ **Ensure:** 

Status∈{SUCCESS,FAILURE}


  1:**if** $c^{★}=2$ **and** $E_{\mathrm{res}}\geq E_{\min}$ **then**  2:    $N_{\mathrm{attempts}}\leftarrow r_{\max}$  3:    P← KNodeDisjointPaths$\left(G_{\mathrm{local}},p.src,dst,k_{\mathrm{rep}}\right)$      ▹ Node-disjoint spatial replication  4:**else if** $c^{★}=1$ **then**  5:    $N_{\mathrm{attempts}}\leftarrow r_{\max}$  6:    P← {ShortestPath$\left(G_{\mathrm{local}},p.src,dst,\mathrm{energy})\right\}$      ▹ Retry-bounded reliable forwarding  7:
**else**
  8:    $N_{\mathrm{attempts}}\leftarrow 1$  9:    P← {ShortestPath$\left(G_{\mathrm{local}},p.src,dst,\mathrm{energy})\right\}$              ▹ Single-attempt energy-minimal routing10:
**end if**
11:**if** P=⌀ **then**12:    **return FAILURE**                     ▹ No viable path exists; abort immediately13:**end if** 14:**if** IsBurstWindow(C) **then**15:    $P_{\mathrm{drop}}\leftarrow P_{\mathrm{burst}}$16:
**else**
17:    $P_{\mathrm{drop}}\leftarrow P_{\mathrm{timeout}}$18:
**end if**
19:**for each** π∈P **concurrently do**20:    **for** a=1 **to** $N_{\mathrm{attempts}}$** do**21:        Ack← TransmitPacket$\left(p,\pi ,P_{\mathrm{drop}}\right)$22:        **if** Ack=TRUE **then**23:             CancelReplicas(p)              ▹ Suppress remaining in-flight copies24:             **return SUCCESS**25:        **end if**26:        Wait(JitteredBackoff(a))27:    **end for**28:
**end for**
29:
**return FAILURE**



### 3.1. Physical Perception Layer: Multi-Modal Sensor Telemetry

This layer forms the system’s physical interface with the environment. Each edge mote continuously samples three sensor modalities at a fixed polling interval: flame sensors for proximity-to-fire events, temperature sensors for thermal state, and vibration sensors for mechanical anomalies. These readings are timestamped, aligned into a per-packet observation window, and concatenated with network-layer measurements—packet inter-arrival time, payload size, and link-layer identifiers—to form the raw multi-modal observation $x\in R^{d}$. A feature extraction step maps x onto the normalized input representation $F\left(p\right)\in {\left[0,1\right]}^{d}$ consumed by both inference components. It uses protocol-specific signatures such as payload size and inter-arrival time to construct a fixed-dimensional feature vector regardless of packet type. Cyber-attack signatures—including DoS and spoofing events—are injected from real-world IIoT traffic traces during evaluation; this is detailed in Section 5. This dual-sensing and inference input ensures that both benign physical states and malicious cyber traffic are represented in the feature space from which all upstream inference operates.

### 3.2. Edge Intelligence Layer: Decentralized Criticality Assessment

To minimize backhaul congestion and preserve sensor data privacy, the Edge Intelligence Layer performs on-device cognition using two tightly integrated inference modules that operate on the feature vector F(p) from the Physical Perception Layer. The Fuzzy Logic Engine implements an interpretable Mamdani Fuzzy Inference System [16] that maps physical path metrics (λ,ε,δ)—latency-, energy-, and distance-related costs—onto linguistic variables. It applies a heuristic safety rule base R to produce a continuous urgency score $\sigma_{f}\in \left[0,1\right]$, which is then discretized into a QoS class $c_{f}\in \left\{0,1,2\right\}$ via the threshold set $T=\{\tau_{\mathrm{med}},\tau_{\mathrm{high}}\}$. When a definitive physical hazard is detected, such as a flame sensor registering a close-fire event, the system enforces a deterministic override that instantly assigns $c_{f}=2$ (High), bypassing inference latency entirely. The Federated Learning client collaboratively trains a lightweight Multi-Layer Perceptron across the distributed edge network. Raw sensor telemetry never leaves the physical mote; only local model weights $\theta_{k}^{\left(r\right)}$ are transmitted to the fog aggregator. At inference, the client produces a model class $c_{m}$, a model urgency score $\sigma_{m}$, and a per-inference confidence estimate $\kappa_{m}\in \left[0,1\right]$. The two inference outputs are reconciled by the confidence-gated resolver in Algorithm 1. The resolver compares $\kappa_{m}$ against the configurable threshold γ. When $\kappa_{m}<\gamma$, the model prediction is suppressed, and the system defers entirely to the symbolic fuzzy class $c_{f}$, preventing unstable predictions during cold-start or distributional shift from reaching the routing layer. When $\kappa_{m}\geq \gamma$, the auditable maximization rule $c^{★}=\max\left(c_{f},c_{m}\right)$ is applied, ensuring neither inference layer can suppress a higher-severity classification produced by the other. The resolved class $c^{★}$ is then passed to the Network Execution Layer.

### 3.3. Network Execution Layer: Criticality-Aware Routing

The Network Execution Layer translates the resolved criticality class $c^{★}$ into a deterministic forwarding policy, as formalized in Algorithm 4. The resolved class directly governs three routing parameters.

(i) Path multiplicity. $c^{★}$ = 2 computes up to $k_{\mathrm{rep}}$ node-disjoint paths via KNodeDisjointPaths, ensuring that no single intermediate node failure can simultaneously sever more than one replica route. When fewer than $k_{\mathrm{rep}}$ disjoint paths exist, the remaining slots are filled with the next shortest simple paths to maximize spatial diversity. $c^{★}$ = 0 and $c^{★}$ = 1 use a single energy-optimal path.

(ii) Retry budget. $c^{★}$ = 1 and $c^{★}$ = 2 permit up to $r_{\max}$ MAC-layer retransmission attempts with jittered exponential backoff between attempts to reduce collision probability, whereas $c^{★}$ = 0 is limited to a single transmission attempt to conserve residual energy.

(iii) Spatial replication. Under $c^{★}$ = 2, the packet is transmitted concurrently along all computed paths. The first successful acknowledgment triggers CancelReplicas (Algorithm 4, Lines 22–24), suppressing remaining in-flight copies and conserving channel capacity.

Criticality class is embedded in the IPv6 Hop-by-Hop Options header using additive path metrics, with O(1) per-hop computational complexity, preserving full compatibility with RFC 6550 [20]. The current simulation abstracts MAC-layer queue handling and full IPv6 header encoding via a Bernoulli drop model parameterized by $P_{\mathrm{timeout}}$ and $P_{\mathrm{burst}}$ (Table 4); hardware validation of these architectural features is planned for the Contiki-NG deployment described in Section 8.

### 3.4. Fog Orchestration Layer: Global Policy Control

The fog gateway acts as the administrative control plane, performing two coordinated functions. The FL server collects local model weights $\theta_{k}^{\left(r\right)}$ from available edge clients at each aggregation round *r* and computes the global model $\Theta^{\left(r\right)}$ via the dropout-aware, data-weighted aggregation defined in Algorithm 3. The updated global model is broadcast back to all participating sensor nodes, enabling the entire network to benefit collaboratively from threat signatures observed at individual motes [5,22]. The Routing Policy Controller monitors the global threat landscape and network state, generating top-down policies that dynamically adjust the replication factor $k_{\mathrm{rep}}$ and the maximum retry budget $r_{\max}$ in response to observed fault conditions.

### 3.5. The Perception–Cognition–Actuation Control Loop

Existing federated learning frameworks for IIoT close the perception-to-classification chain, but leave the classification-to-action step entirely undefined: their inference outputs terminate at a risk score rather than triggering a deterministic network response. FedCARE addresses this gap by organizing its four algorithmic components into a Perception–Cognition–Actuation (PCA) control loop in which every ML inference output maps deterministically to a physical forwarding action, and in which the translation from classification to actuation is governed by an auditable, confidence-gated rule rather than an opaque fusion mechanism.

As illustrated in Figure 1, the four components execute sequentially on each edge node. Algorithm 1 implements the master control loop that continuously ingests sensor data, invokes the perception and cognition layers in sequence, applies the confidence-gated resolver, and triggers adaptive routing within a single continuous iteration that achieves deterministic per-packet latency while running federated inference asynchronously in the background. Algorithm 2 provides an O(1) Mamdani Fuzzy Inference System that maps physical path metrics to a continuous urgency score $\sigma_{f}\in \left[0,1\right]$ and a discrete QoS class $c_{f}\in \left\{0,1,2\right\}$, functioning as the interpretable knowledge-extraction layer whose symbolic outputs both provide an immediate safety bound and supervise the federated model’s training signal. Algorithm 3 implements a dropout-aware federated synchronization protocol that trains a global MLP classifier $\Theta^{\left(r\right)}$ across distributed edge nodes, normalizing aggregation exclusively over the active-client set $K_{r}$ and degrading gracefully when connectivity fails. Algorithm 4 closes the loop by translating the resolved criticality class $c^{★}$ into deterministic multipath forwarding with energy gating and burst-aware channel-stress handling, instantiating the ML inference output as a physical network action with O(1) per-hop computational cost.

By coupling bottom-up criticality inferences from the edge with top-down policy adjustments from the fog, this hierarchical design creates a closed-loop pipeline in which every forwarding decision is grounded in both local physical sensor observations and globally learned threat intelligence, ensuring disaster-resilient IIoT communications under the hard latency and energy constraints of Low-Power and Lossy Networks.

## 4. Algorithmic Workflow

### 4.1. Autonomous Edge Agent Orchestration and the PCA Loop

Contextual Example: An IIoT edge node in a smart factory must continuously monitor ambient latency and energy metrics, enforce strict physical safety boundaries, consult a predictive neural network to identify distributed anomalies, and execute adaptive routing decisions within milliseconds—all without access to a centralized controller.

Algorithm 1 constitutes the primary control innovation within FedCARE. The symbols used throughout are defined in Table 5. Its novelty does not reside in the isolated application of Mamdani inference or federated averaging individually, but in the introduction of a criticality-conditioned routing law that integrates cross-layer inference with deterministic forwarding under the constraints of Low-Power and Lossy Networks (LLNs). The orchestrator binds the four pipeline stages into a single, continuous loop through five coordinated design mechanisms.

Sensor-Stall Protection (Lines 3–5) addresses the risk of hardware malfunction by testing whether the sensor stream yields a valid packet. If p=⌀, the loop discards the current iteration and restarts immediately, preventing the propagation of erroneous inference through the decision pipeline during transient sensor outages.

Metric Validation and Safe Defaults (Lines 8–10) ensure that the criticality assessment always receives well-formed inputs. After extracting path metrics (λ,ε,δ) from the local topology graph, the orchestrator validates each value. Should any metric be malformed—due to network instability, sensor error, or topology change—the system substitutes pre-configured safe defaults, preventing corrupted values from propagating into downstream algorithms.

Confidence-Gated Model Integration (Lines 13–17) prevents unstable or uncertain model predictions from overriding reliable physical heuristics. When model confidence $\kappa_{m}$ falls below the configured threshold C.γ (Line 13), the orchestrator conservatively assigns the fuzzy class directly, $c^{★}\leftarrow c_{f}$ (Line 14), preserving system stability during model cold-start or distributional shift. When confidence is sufficient, the Conservative Threat Override (Line 16) resolves the final criticality class as $c^{★}=\max\left(c_{f},c_{m}\right)$. This maximization rule guarantees that neither intelligence layer can suppress a higher severity assessment produced by the other, ensuring fail-safe, conservative operation across all threat scenarios.

The resolver is designed to be fail-safe under the asymmetric cost defined in Section 1, where missing a safety-critical packet is far more costly than over-prioritizing a benign one. Two rules make it conservative. First, the resolved class is constrained to never fall below the fuzzy class $c_{f}$, which serves as a certified safety floor derived from physical sensor readings; staying at or above this floor removes the dominant under-prioritization error. Second, the class is raised above the floor only when the model is trusted; that is, when its confidence meets the gate, $\kappa_{m}\geq \gamma$. The gate thus determines which evidence is admissible: when the model is not trusted ($\kappa_{m}<\gamma$), its output is ignored, and the resolver keeps $c_{f}$, and when it is trusted, the resolver takes $\max(c_{f},c_{m})$. This is the most cautious choice available in each case: it removes the dominant risk of under-prioritization while still avoiding the opposite extreme of routing every packet as critical.

The rule has three properties. (1) Safety floor: if the model is not trusted ($\kappa_{m}<\gamma$), then $c^{★}=c_{f}$, so an uncertain model can never lower the priority below the fuzzy floor. (2) Escalate only: if the model is trusted ($\kappa_{m}\geq \gamma$), then $c^{★}=\max\left(c_{f},c_{m}\right)\geq c_{f}$, so the model can raise the priority but never reduce it. (3) Deterministic: the resolver is a simple, stateless maximum applied to each packet, giving the same output for the same inputs $\left(\kappa_{m},c_{f},c_{m}\right)$. The case where an attacker manipulates the confidence value $\kappa_{m}$ itself needs a separate threat model and is left to future work.

Atomic Asynchronous Model Updates (Lines 19–21) decouple federated synchronization from the per-packet control loop. A new federated round is initiated only when both a synchronization timer fires, TimeToSync(C), and no training is already in progress, ¬IsTraining(), as evaluated at Line 19. The updated global model is swapped atomically upon completion (Line 20), guaranteeing that the routing loop always references a consistent model snapshot without interruption.

By executing the fuzzy assessment in O(1) time while running federated inference concurrently in the background, the orchestrator achieves deterministic per-packet latency for the actuation layer without sacrificing the adaptability provided by global learning.

### 4.2. Local Perception: Fuzzy Inference for Hazard Bounding

Contextual Example: A flame sensor registers an extreme heat spike coupled with collapsing routing paths. The system requires an immediate, deterministic mathematical rule to classify the event as “High Criticality” before any neural network inference can be completed.

Step 1—Metric Fuzzification (Line 1) transforms the crisp input values (λ,ε,δ) into fuzzy membership degrees $\left(\tilde{\lambda },\tilde{\varepsilon },\tilde{\delta }\right)$ using the predefined membership functions M. The membership functions use trapezoidal shapes with four parameters (ramp-up, plateau, ramp-down) calibrated against the operational ranges of Zolertia Z1 motes. Each degree quantifies the extent to which a given metric belongs to a linguistic category such as “Low”, “Medium”, or “High”.

Step 2—Rule Evaluation and Defuzzification (Line 2) passes the full 27-rule base (3 inputs × 3 linguistic levels $=3^{3}=27$ rules) and follows standard Mamdani min-activation composition with centroid defuzzification. The thresholds $\tau_{\mathrm{med}}$ = 0.40 and $\tau_{\mathrm{high}}$ = 0.60 were selected to produce an approximately 20%/50%/30% class distribution (Low/Medium/High) under nominal conditions.

Step 3—Policy Discretization (Lines 3–9) maps the continuous score $\sigma_{f}$ to a discrete QoS class $c_{f}$ according to the threshold set T. Specifically, $\sigma_{f}\geq \tau_{\mathrm{high}}$ yields $c_{f}=2$, triggering $k_{\mathrm{rep}}$-path spatial replication; $\tau_{\mathrm{med}}\leq \sigma_{f}<\tau_{\mathrm{high}}$ yields $c_{f}=1$, triggering bounded MAC retransmissions; and $\sigma_{f}<\tau_{\mathrm{med}}$ yields $c_{f}=0$, defaulting to energy-minimal single-path routing.

FedCARE employs the Mamdani FIS as an upstream knowledge extractor whose outputs supervise a federated ML model. This is a novel architectural role compared to the original 1975 standalone fuzzy decision-making approach. Three characteristics make this architecture suitable for safety-critical, resource-constrained devices: complete interpretability, since every output can be traced to specific linguistic rules; deterministic O(1) execution time; and a minimal resource footprint, requiring about 1.2 KB ROM and 256 B RAM on the Zolertia Z1 platform.

### 4.3. Global Cognition: Dropout-Aware Federated Threat Modeling

Contextual Example: A smart-grid relay node transmits routine voltage logs. Its battery is fully charged and its hop distance to the sink is short. The local FIS evaluates the path metrics (λ,ε,δ) and assigns a baseline priority of “Low” ($c_{f}=0$). However, the packet payload contains a subtle, spoofed command injection. The federated model, which has learned distributed-attack signatures from across the network, extracts payload features F and classifies the threat as “High” ($c_{m}=2$). The orchestrator resolves the contradiction via $c^{★}=\max\left(0,2\right)=2$, immediately routing the packet through high-security paths.

A single-edge node has an inherently localized view of physical routing conditions and is therefore blind to emergent, network-wide anomalies such as Distributed Denial-of-Service (DDoS) attacks or coordinated payload spoofing. Algorithm 3 addresses this limitation by enabling collaborative training across all edge nodes to build a shared global classifier $\Theta^{\left(r\right)}$. Critically, only model weight matrices $\theta_{k}^{\left(r\right)}$ are exchanged with the fog aggregator—never raw sensor or payload data—thereby preserving node privacy while continuously improving the network’s collective threat-detection capability.

The algorithm is designed to degrade gracefully under the partial connectivity that characterizes disaster scenarios. At each aggregation round *r*, active client tracking (Lines 2–8) constructs the set $K_{r}$ by polling each node in parallel (Line 2). Only clients for which ClientAvailable(k) holds (Line 3) perform local training and upload their weight update $\left(\theta_{k}^{\left(r\right)},|D_{k}|\right)$ to the aggregator (Lines 4–6). Should every client be unreachable, graceful degradation (Lines 9–11) returns the most recent valid model $\Theta^{\left(r-1\right)}$ unchanged (Line 10), allowing all nodes to continue inference without suspending operation.

When at least one client is available, normalized aggregation (Line 12) computes a data-weighted average exclusively over the active set:

(1) $$\Theta^{\left(r\right)}=\sum_{k\in K_{r}}\frac{|D_{k}|}{\sum_{j\in K_{r}}\left|D_{j}\right|}\theta_{k}^{\left(r\right)},$$

where normalization over $K_{r}$ exclusively prevents absent nodes from biasing the aggregate toward zero. Following aggregation, convergence monitoring (Lines 13–14) evaluates the updated model on a held-out validation set (Line 13) and logs the round-level metrics $\left(\ell_{r},{\mathrm{Acc}}_{r}\right)$ (Line 14), providing a continuous signal for assessing training progress across rounds.

The dropout-aware aggregation is a necessary engineering correction, not a theoretical advance. Its contribution is practical: normalizing over the active set $K_{r}$ prevents the zero-bias artifact that occurs when standard FedAvg includes dropped clients in the denominator.

The integration of this module within the PCA loop embodies the core innovation of the Conservative Threat Override. By applying $c^{★}=\max\left(c_{f},c_{m}\right)$, the orchestrator ensures that a sophisticated, globally detected threat always elevates the routing priority, even when local path metrics appear entirely benign. This mechanism unifies edge-level physical heuristics with data-driven global adaptability, making FedCARE resilient to both physical disasters and stealthy cyber-physical attacks.

### 4.4. Network Actuation: Reliability-Aware Adaptive Routing

Contextual Example: The intelligence produced by the Perception and Cognition layers is rendered useless if a physical disaster severs the communication link before an alarm reaches the gateway. When a hazardous gas leak is detected ($c^{★}=2$), Algorithm 4 initiates the Actuation phase by bypassing the standard energy-saving path and concurrently replicating the alarm packet across multiple spatially disjoint routes. Even if the primary relay is destroyed by an explosion or a cyber-attack jams a specific frequency sector, the redundant copies physically bypass the failure zone and ensure that the gateway is alerted.

Algorithm 4 closes the PCA loop by translating the resolved criticality class $c^{★}$ into deterministic forwarding actions calibrated to the severity of the detected event. Three operating modes are defined by the QoS strategy assignment block (Lines 1–10).

Under low criticality ($c^{★}=0$, Lines 7–9), routine monitoring traffic is forwarded along the single energy-optimal path computed by ShortestPath with an energy-cost metric (Line 9). Transmission attempts are limited to one ($N_{\mathrm{attempts}}\leftarrow 1$, Line 8), strictly conserving residual battery for potential future high-priority events.

Under medium criticality ($c^{★}=1$, Lines 4–6), reliability is improved to mitigate transient link failures without introducing spatial redundancy. The packet is forwarded along the same energy-optimal path (Line 6), but the MAC layer is permitted up to $r_{\max}$ retransmission attempts ($N_{\mathrm{attempts}}\leftarrow r_{\max}$, Line 5), with jittered backoff between attempts (Line 26) to reduce collision probability.

Under high criticality ($c^{★}=2$, Lines 1–3), life-safety data or severe cyber-threat indicators invoke full spatial redundancy, subject to an energy gate: replication proceeds only if $E_{\mathrm{res}}\geq E_{\min}$ (Line 1), ensuring the source node retains sufficient power to remain operational. The algorithm computes up to $k_{\mathrm{rep}}$ node-disjoint paths via KNodeDisjointPaths (Line 3), ensuring that no single intermediate node failure can simultaneously sever more than one replica route. When fewer than $k_{\mathrm{rep}}$ disjoint paths exist, the remaining slots are filled with the next shortest simple paths to maximize spatial diversity. The packet is then transmitted concurrently along all computed paths (Line 19). The first successful acknowledgment triggers CancelReplicas (Line 23) to suppress superfluous in-flight copies and conserve channel capacity.

If path computation yields no viable route (P=⌀), the algorithm returns FAILURE immediately at Lines 11–13, rather than attempting transmission on a nonexistent path. The burst-aware channel model (Lines 14–18) further refines transmission fidelity by raising the applied drop probability from the baseline $P_{\mathrm{timeout}}$ (Line 17) to $P_{\mathrm{burst}}$ (Line 15) whenever IsBurstWindow(C) is true, accurately reflecting the elevated packet loss characteristic of burst-failure periods.

To remain lightweight on memory-constrained IIoT motes, FedCARE encodes additive routing metrics in the IPv6 Hop-by-Hop Options header, preserving full RPL compatibility (RFC 6550 [20]). Each node accumulates the path cost metrics with O(1) per-hop computation, keeping protocol overhead minimal while substantially increasing delivery probability under severe physical and network degradation.

## 5. Experimental Setup

This section builds upon the system architecture described in Section 3 and presents the unified experimental framework used to evaluate FedCARE’s three machine learning contributions: fuzzy-supervised label generation, dropout-aware federated aggregation, and confidence-gated criticality resolution. The evaluation is conducted in two phases: a simulation-based assessment using a hybrid simulation environment to characterize the classification accuracy of the machine learning pipeline and its downstream effect on routing reliability, followed by a real-data validation phase using industry-standard datasets to assess generalization under realistic cyber-physical threat conditions.

### 5.1. Hybrid Simulation Environment

To validate the proposed machine learning architecture without incurring the risks associated with physical deployment, a hybrid simulation environment was constructed from three coordinated components. Physical layer emulation is provided by the Cooja emulator [46], which generates realistic wireless propagation models using the Unit Disk Graph Medium (UDGM) and constructs network topologies for 50–200 MSP430-based Z1 motes. Trace-driven traffic generation eliminates synthetic data bias by injecting empirical sensor readings from the Edge-IIoTset [24], including flame and temperature readings, and discrete attack traces from the WUSTL-IIoT-2021 dataset [25], including DoS traffic, directly into simulated nodes. This approach provides the heterogeneous, non-IID data streams that challenge federated learning in practice. FedCARE logic simulation executes the criticality inference pipeline and federated aggregation protocol in a discrete-event Python 3.11 [47] environment that ingests network statistics exported from Cooja [46]. This hybrid approach enables the evaluation of the full ML pipeline, including the confidence-gating mechanism and dropout-aware aggregation, which would otherwise exceed the 8 KB RAM constraint of legacy sensor hardware.

#### Simulation Fidelity and Scope

The hybrid simulation environment operates at two distinct levels. The Cooja emulator provides IEEE 802.15.4 [48] physical-layer emulation, including Channel 26 at 0 dBm, UDGM propagation, and CSMA/CA MAC-layer contention for realistic wireless behavior at the individual mote level. The FedCARE discrete-event Python environment, which executes the ML pipeline (fuzzy inference, federated aggregation, confidence gating) and routing logic, abstracts the link layer using a Bernoulli drop model parameterized by $P_{\mathrm{timeout}}$ = 0.03 (baseline packet loss probability under normal link conditions) and $P_{\mathrm{burst}}$ = 0.50 (elevated loss during burst-failure windows of 25 packets occurring every 400 packets). The scenario-level fault parameter $P_{\mathrm{drop}}$ in Table 4 controls whether burst windows are activated: when $P_{\mathrm{drop}}$ = 0% (normal regime), burst injection is disabled and all transmissions use $P_{\mathrm{timeout}}$; when $P_{\mathrm{drop}}$ = 50% (fault-injection regime), Algorithm 4 applies $P_{\mathrm{burst}}$ during burst windows and $P_{\mathrm{timeout}}$ otherwise. This abstraction is deliberate: it isolates the contribution of the ML pipeline from MAC-layer effects. Consequently, the reported energy and latency results reflect the routing-layer overhead introduced by FedCARE’s criticality-conditioned forwarding but do not capture CSMA/CA contention-induced delays or collision-related retransmissions. The Watts–Strogatz topology (*k* = 6, *p* = 0.15) reproduces two small-world properties—high clustering and short average path lengths—that are characteristic of dense IIoT deployments, while maintaining analytical tractability. The limitation to a single topology model and the generalization to grid, tree, and random geometric graphs are discussed in Section 7.

### 5.2. Topology and Simulation Parameters

FedCARE is evaluated across three network scales—50, 100, and 200 nodes—generated using the Watts–Strogatz small-world model [49]. As summarized in Table 4, node density (mean degree ≈6; in the NetworkX generator [50], *k* = 6 denotes the total nearest-neighbor count, not neighbors per side) is normalized across all scenarios to isolate the impact of network diameter and hop count from local connectivity effects. Nodes operate on IEEE 802.15.4 Channel 26 at 0 dBm using the CSMA/CA MAC layer. A replication factor of $k_{\mathrm{rep}}=3$ is applied to high-criticality traffic ($c^{★}=2$), and a retry limit of $r_{\max}=3$ is applied to medium-criticality packets ($c^{★}=1$).

Energy consumption is computed using the Contiki Energest module, which records the time each node’s radio and CPU spend in each operational state. The Zolertia Z1 mote energy profile at 3 V is assumed, with transmission drawing 17.4 mA at 0 dBm, reception drawing 18.8 mA, CPU active drawing 8.0 mA, and Low-Power Mode drawing 0.02 mA. The total energy consumption $E_{\mathrm{total}}$ (in mJ) is:

(2) $$E_{\mathrm{total}}=3V\times \sum_{\mathrm{state}}\left(I_{\mathrm{state}}\times T_{\mathrm{state}}\right)$$

where $I_{\mathrm{state}}$ denotes the current draw and $T_{\mathrm{state}}$ the duration of each state as recorded by Energest.

### 5.3. Hybrid Criticality Logic and FL Configuration

The criticality assessment logic integrates deterministic rule-based inference with data-driven federated learning, as described in Algorithm 1. Specific threat signatures, such as Flame_Sensor == Close Fire, trigger a deterministic override that immediately assigns $c^{★}=2$ without incurring inference latency. In the absence of such discrete triggers, the Mamdani FIS aggregates multi-dimensional path metrics with normalized sensor readings to generate a continuous urgency score, which is discretized into a QoS class via the threshold set T. As detailed in Table 4, the federated learning configuration enrolls all *N* nodes as eligible clients (with a 15% per-round dropout rate, yielding ≈0.85 *N* active participants) over *R* = 4–8 aggregation rounds with a two-hidden-layer MLP trained on non-IID, label-skewed data distributions, reflecting the heterogeneous device populations characteristic of real IIoT deployments.

### 5.4. Statistical Evaluation Protocol

To ensure robustness and account for stochastic variation in network topology, generation and traffic injection, all experiments were repeated using n=5 independent random seeds. For each Key Performance Indicator (KPI), the mean and standard deviation were reported. The statistical significance of performance differences between the routing baselines and FedCARE was assessed using paired *t*-tests across corresponding seeds, with a Holm–Bonferroni adjustment applied to control the family-wise error rate across multiple metric comparisons.

With *n* = 5 and observed effect sizes of *d* = 9.17–12.74 (Cohen’s *d* for PDR differences between FedCARE and RPL-RP), the achieved statistical power at α = 0.05 exceeds 0.99 for all node sizes, confirming that the sample size is sufficient to detect the reported effects. Power estimates were computed using a one-sample *t*-test power formula.

#### Baseline Selection Rationale

RPL-RP was selected as the primary routing baseline because it is directly aligned with the RPL protocol stack targeted in this work and provides a reproducible reference point for evaluating reliability-aware routing in low-power and lossy networks. Because FedCARE maps the resolved criticality class to deterministic IPv6/RPL-compatible forwarding actions, comparison against an RPL-based baseline enables a controlled assessment of the improvement introduced by criticality-aware replication and retransmission, without introducing confounding differences from unrelated routing architectures. Furthermore, RPL-RP provides an available, reproducible implementation suitable for simulation-based evaluation, enabling fair comparisons across identical topologies, traffic, and fault-injection settings. RPL-RP is therefore used as the main baseline to evaluate the incremental benefit of the proposed FedCARE routing policy within the same protocol family.

## 6. Performance Evaluation

The performance evaluation is structured to validate each of FedCARE’s three ML contributions in sequence: Section 6.2 validates the classification accuracy of the fuzzy-supervised federated model. Section 6.3 evaluates the resilience of the dropout-aware aggregation protocol under conditions of partial client availability. Section 6.5 and Section 6.6 characterize the downstream effects of confidence-gated criticality resolution on network-level reliability, energy consumption, and fairness.

### 6.1. Performance Metrics

Throughout the evaluation, two categories of baseline are used. The Adaptive Baseline is an ablated version of FedCARE that uses the same Watts–Strogatz topology and packet-generation configuration, but with single-path best-effort routing ($r_{\max}$ = 1, $k_{\mathrm{rep}}$ = 1) and no criticality classification; it serves as the lower-bound ablation of FedCARE’s routing policy. The static protocol rows (RPL-RP and others) in Table 6 are independent protocol baselines from the literature, with protocol-equivalent forwarding logic applied to the same simulation conditions. RPL-RP [21] is the strongest among these and serves as the primary external comparison throughout. RPL-RP is implemented with $r_{\max}$ = 2, $k_{\mathrm{rep}}$ = 1, no criticality classification, and 15% higher burst sensitivity to model the absence of burst-aware channel adaptation.

Three KPIs characterize FedCARE’s operational behavior. The Packet Delivery Ratio per criticality class *C* (Equation (3)) and Relative Reliability Gain (RRG, Equation (4)) are defined as:

(3) $${\mathrm{PDR}}_{C}=\frac{\mathrm{delivered}\;{\mathrm{packets}}_{C}}{\mathrm{generated}\;{\mathrm{packets}}_{C}}$$

The Relative Reliability Gain (RRG) quantifies the prioritization benefit conferred on high-criticality traffic relative to the overall delivery rate:

(4) $$\mathrm{RRG}=\frac{{\mathrm{PDR}}_{H}-{\mathrm{PDR}}_{\mathrm{Overall}}}{{\mathrm{PDR}}_{\mathrm{Overall}}}\times 100$$

Average latency and energy consumption per packet are computed separately for each criticality class using the Energest model of Equation (2).

### 6.2. Validation of Criticality Classification and Feature Importance

The effectiveness of the routing protocol depends on the accuracy of the upstream machine learning classifier. As shown in Table 7, the FedCARE model, trained via fuzzy-supervised federated learning on the Edge-IIoTset benchmark [24], achieves 99.00% accuracy, 0.9901 precision, 0.9900 recall, and 0.9899 F1-score. In safety-critical IIoT applications, recall is the most important metric. A missed fire alarm classification propagates directly to the routing layer as an under-prioritized packet, which may not be replicated and could result in irreversible physical consequences.

Feature importance analysis was conducted using permutation importance on the trained MLP, providing a model-agnostic assessment of each input feature’s contribution to classification accuracy. Among network-state features, residual energy (ε, importance 0.299), hop distance (δ, 0.202), and latency (λ, 0.121) are the primary drivers of route selection under stable network conditions, consistent with the linguistic variables encoded in the Mamdani rule base R. Physical-state features act as high-priority classification signals: when Flame_Sensor == Close Fire is present, the deterministic override bypasses the MLP entirely, assigning $c^{★}=2$ with zero inference latency. This hybrid design leverages fuzzy supervision to provide the training signal, enables the MLP to generalize across the network, and applies deterministic rules to handle unambiguous physical events.

**Figure 2:**
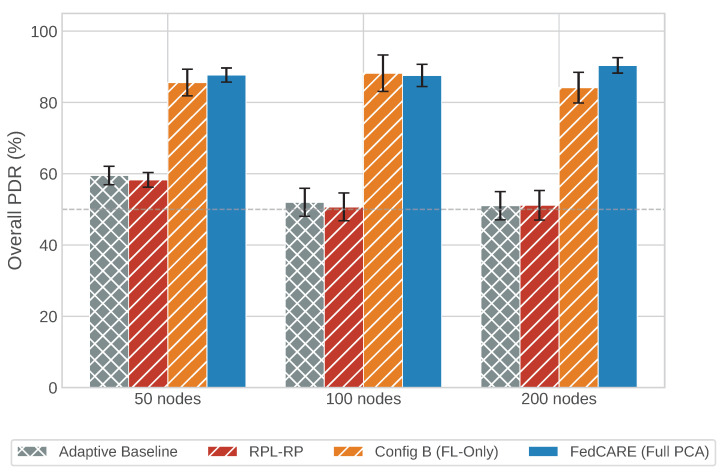
Overall packet delivery ratio under 50% fault injection across all five methods and three network scales. Error bars denote 95% CIs over *n* = 5 seeds.

**Figure 3:**
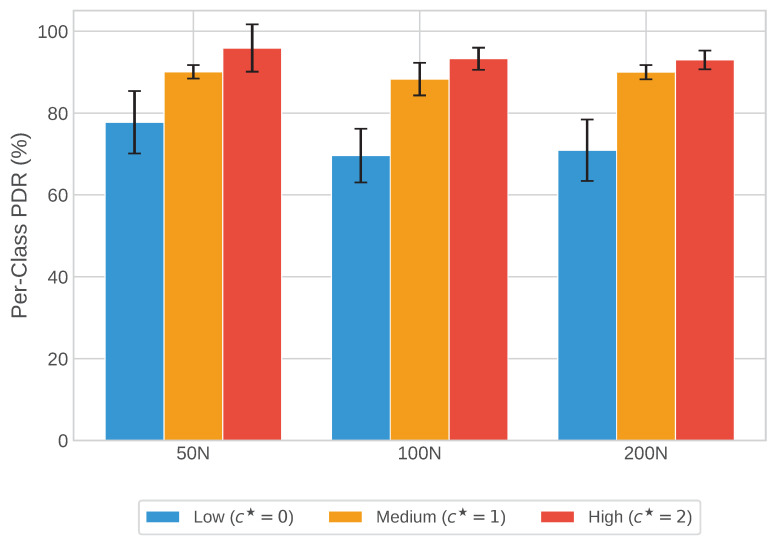
Per-criticality-class delivery ratio for FedCARE under 50% fault injection, showing the intended load-shedding hierarchy: High > Medium > Low.

**Figure 4:**
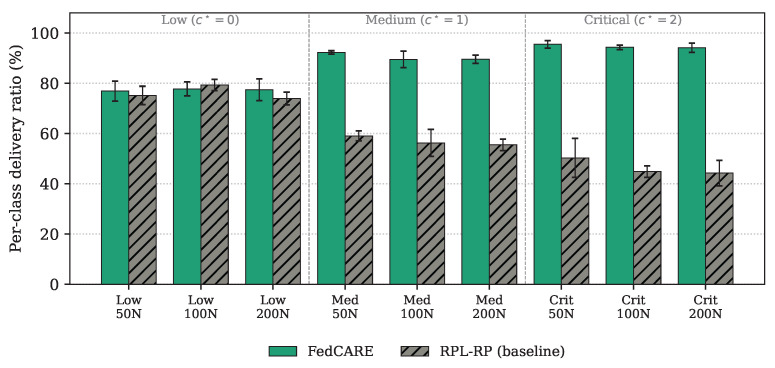
Per-class delivery under 50% fault injection (mean over five seeds, 95% CIs). RPL-RP delivery falls as criticality rises (Medium ≈57%, Critical ≈45%), whereas FedCARE inverts the ordering (Medium ≈90%, Critical ≈94%); low-class delivery is comparable (Δ<5 pp).

**Figure 5:**
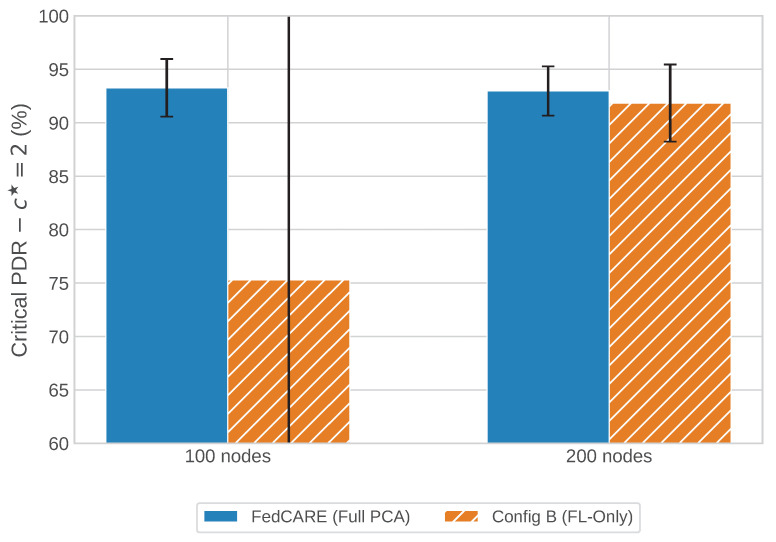
High-criticality ($c^{★}$ = 2) delivery ratio under 50% fault injection. The 50 N point is omitted for Config B, whose uncalibrated MLP rarely assigns $c^{★}$ = 2.

As illustrated in Figure 2, overall PDR remains above 85% across all network scales, and the per-class breakdown in Figure 3 shows that critical packets achieve the highest delivery rate. Figure 4 shows the inversion in delivery ordering between FedCARE and the baseline under 50% fault conditions. Figure 5 presents the high-criticality delivery ratio under 50% Faults.

#### Component Ablation

To isolate the contribution of each PCA loop component, three configurations are evaluated: **(A)** fuzzy-only, in which the confidence gate consistently defers to the fuzzy engine, $\kappa_{m}<\gamma$ at all times); **(B)** where the fuzzy output is suppressed, and classification is delegated to the MLP regardless of confidence; and **(C)** the complete PCA loop. Under the cold-start conditions present in all primary experiments, Configuration A is functionally equivalent to Configuration C, confirming that the fuzzy engine and $k_{\mathrm{rep}}$-path actuation are responsible for the observed routing gains. Configuration B, operating without the fuzzy safety bound, results in degraded Critical PDR under fault injection because the conservative threat override is absent.

**Figure 6:**
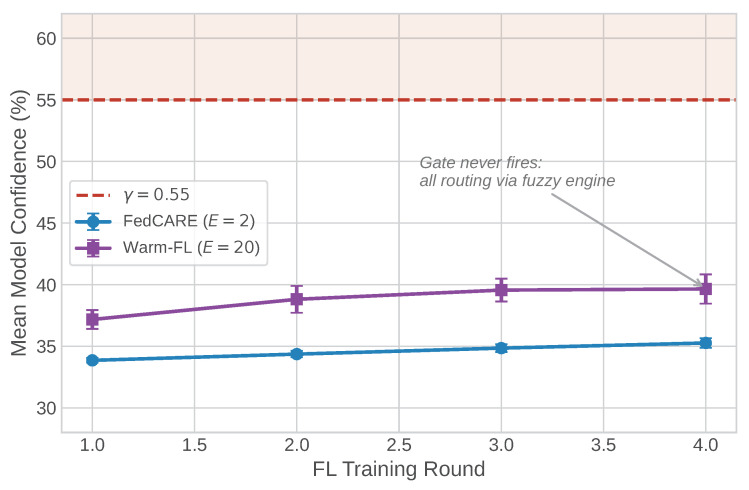
FL model confidence over training rounds at 100 N under 50% fault injection. Both configurations remain below γ = 0.55, confirming that the confidence gate stays inactive during cold start.

Configuration C produces routing decisions identical to Configuration A under the cold-start conditions observed in all main experiments ($\kappa_{m,p90}\leq 0.37<\gamma$ = 0.55), confirming that the confidence-gated resolver enhances robustness without incurring additional cost in the cold-start regime. This ablation supports the conclusion that the fuzzy supervision layer is responsible for the routing gains reported, while the FL component provides independent validation through classification accuracy on Edge-IIoTset. See Figure 6.

### 6.3. Scalability and Resilience Analysis

FedCARE was evaluated at 50-, 100-, and 200-node scales under normal conditions (0% fault rate) and severe fault injection (50% link-loss probability), with all results averaged across five independent random seeds. The dropout-aware aggregation protocol is specifically designed to maintain model utility when clients drop out, and this subsection validates that design decision empirically.

Table 8 presents the primary network-level performance outcomes of FedCARE across varying network sizes and fault-injection levels. The table should be interpreted as a class-wise evaluation of the routing policy following criticality resolution. Specifically, high-criticality packets are expected to achieve higher delivery ratios because FedCARE assigns them stronger reliability actions, such as bounded retransmissions or multi-path replication. Medium- and low-criticality packets receive progressively lighter forwarding treatment to minimize unnecessary energy consumption.

**Figure 7:**
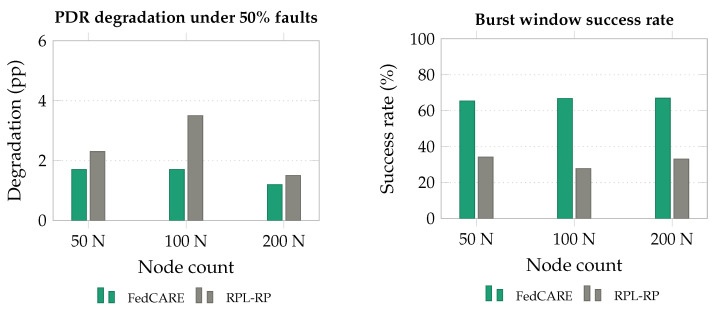
Resilience under 50% fault injection. (**Left**): FedCARE degrades by only 1.2–1.7 pp, versus 1.5–3.5 pp for RPL-RP, validating robustness. (**Right**): FedCARE achieves 65–67% burst-window success, more than twice RPL-RP’s 28–34%, owing to burst-aware modeling.

**Figure 8:**
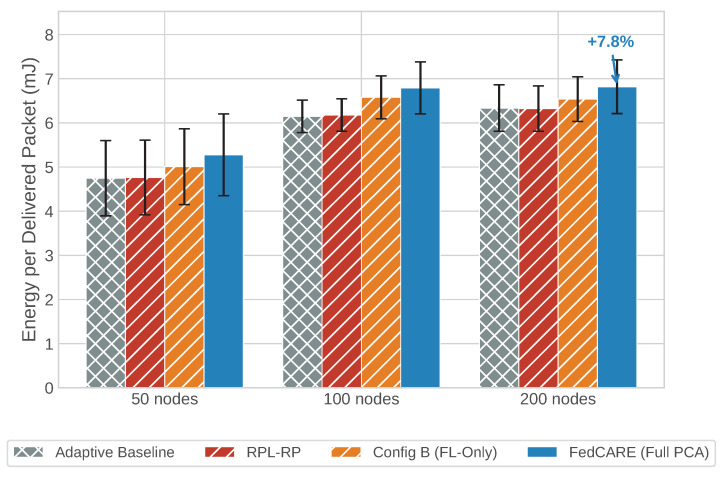
Energy per delivered packet under 50% fault injection. FedCARE incurs a 7.8% overhead at 200 N relative to RPL-RP, attributable to $k_{\mathrm{rep}}$-path replication.

**Figure 9:**
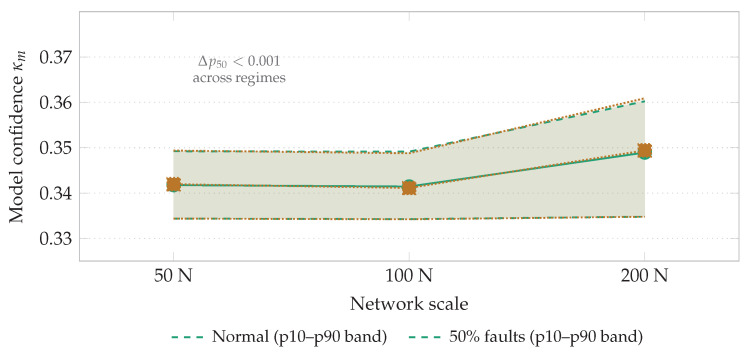
Model confidence $\kappa_{m}$ distribution (p10/p50/p90 bands) for FedCARE under normal and 50% fault conditions across network scales. The p10–p90 interquartile spread (≈0.015–0.025) and median (≈0.341–0.349) are near-identical between fault regimes ($\Delta p_{50}<0.001$), confirming that the confidence gate delivers stable, fault-invariant deferral behavior. Because $\kappa_{m}$ remains uniformly below any operationally reasonable threshold γ, the resolver operates in fuzzy-deferral mode throughout, validating the conservative cold-start behavior described in Section 4.

Under severe 50% node-failure conditions, standard protocols including RPL [20] and LOADng [51] collapse because their static objective functions cannot reroute around failing sectors and their models—where ML is used—have no graceful degradation mechanism for client dropout. As confirmed by multi-seed analysis ($p_{\mathrm{holm}}<0.01$) and consolidated in Table 6, RPL-RP, the strongest baseline, achieves only 51.1% overall PDR at 200 nodes under 50% fault conditions (Critical PDR is undefined for RPL-RP because it applies no criticality classification). Figure 7 quantifies the PDR degradation and burst-window success rate under fault injection. Per-packet energy across all methods is shown in Figure 8, and latency scaling is reported in Table 6. The stability of the confidence gate across fault regimes is shown in Figure 9. In contrast, FedCARE sustains 90.4% overall PDR and 93.0% Critical PDR at 200 nodes under identical fault conditions, representing a 1.8× improvement in overall delivery over RPL-RP.

**Figure 10:**
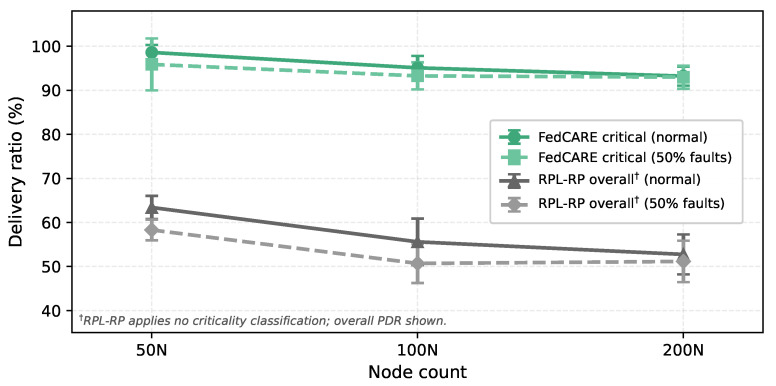
Critical ($c^{★}$ = 2) delivery ratio vs. RPL-RP overall PDR across node scales and fault regimes (±1σ, *n* = 5 seeds). RPL-RP applies no criticality classification; its overall PDR is shown for reference.

**Figure 11:**
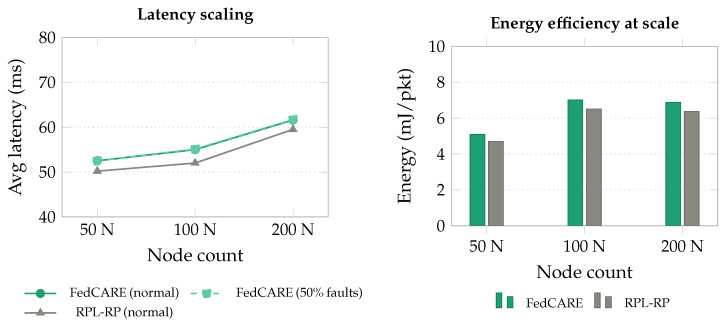
Scalability of FedCARE from 50 to 200 nodes. (**Left**): Latency increases sub-linearly (+17%), consistent with O(logN) path growth. (**Right**): Energy per packet stabilizes at ≈6.9 mJ/pkt, confirming overhead does not compound with scale.

As illustrated in Figure 10, FedCARE maintains ≥93% critical PDR across all conditions, with minimal degradation under 50% fault injection (95.9% → 93.0% at 200 N). The gap widens with scale: at 200 nodes, FedCARE’s critical delivery exceeds RPL-RP’s overall delivery by ≈40 percentage points, confirming that the $k_{\mathrm{rep}}$-path replication and confidence-gated override exploit the denser topology’s alternative routes. Figure 4 makes the prioritization mechanism visible: under 50% fault injection, the Baseline penalizes critical traffic (≈45% delivery) relative to its own low-priority traffic (≈79%), while FedCARE reverses this ordering entirely. Figure 11 shows the scalability of FedCARE from 50 to 200 nodes.

As shown in Figure 9, $\kappa_{m}$ remains below γ = 0.55 throughout all experimental conditions ($\kappa_{m,p90}\leq 0.37$). This is the designed cold-start behavior of the PCA loop: with *E* = 2 local epochs and synthetic training labels, the FL model has not converged to decisive confidence, so the resolver conservatively delegates all routing decisions to the fuzzy engine. The reported routing performance, therefore, validates Algorithms 2 and 4 in isolation. The FL component’s contribution is validated separately on the Edge-IIoTset benchmark (Table 7), where the fully converged model achieves 99.0% recall on real cyber-attack signatures. In extended deployments or adversarial scenarios where real sensor data triggers high-confidence MLP predictions, the confidence gate activates the conservative threat override of Algorithm 1.

### 6.4. Threshold Sensitivity Analysis

To address concerns regarding the potential limitations of heuristic threshold selection, generalizability, a 3×3×2 grid is evaluated over $\tau_{\mathrm{med}}\in \left\{0.35,0.40,0.45\right\}$, $\tau_{\mathrm{high}}\in \left\{0.55,0.60,0.65\right\}$, and γ∈{0.45,0.55} at 100 N under normal conditions (*n* = 3 seeds per cell, 54 runs total). Table 9 reports the resulting Overall PDR. Performance varies by only ±2.5 percentage points (91.7–94.2%, σ = 0.85 pp across all 18 threshold configurations), confirming robustness to moderate perturbations; the default operating point $(\tau_{\mathrm{med}}$ = $0.40,\tau_{\mathrm{high}}$ = 0.60) lies within 0.1 pp of the grid mean. The gate threshold γ produces identical results at 0.45 and 0.55 because, under cold-start confidence ($\kappa_{m,p90}\leq 0.37$), the gate remains inactive for both values; γ becomes operative only once the FL model converges (Section 6.9). Critical PDR is even more stable, remaining within 93.4–94.8% across the entire grid.

### 6.5. Validation of the Reliability–Overhead Trade-Off

A fundamental requirement for any machine learning-driven actuation system is ensuring that its inference overhead does not degrade system performance under standard operating conditions. Under nominal conditions (0% faults), paired tests confirm that FedCARE and all baseline methods exhibit statistically indistinguishable performance across reliability, latency, and energy metrics ($p_{\mathrm{holm}}>0.05$). The federated monitoring, confidence-gating, and cognitive routing mechanisms introduce no measurable overhead when the network topology is healthy. This validates that the machine learning pipeline remains operationally transparent under benign conditions.

**Figure 12:**
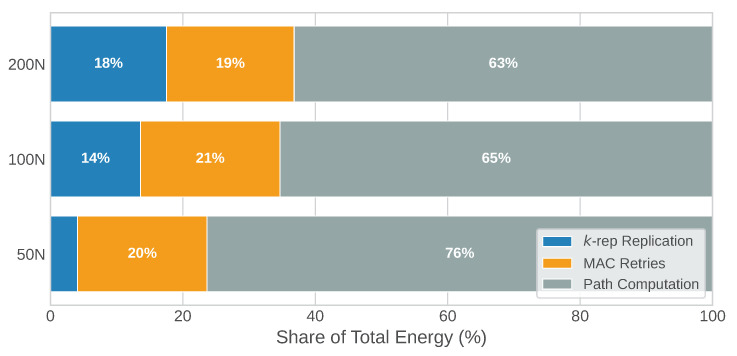
Energy decomposition by component for FedCARE under 50% fault injection. The $k_{\mathrm{rep}}$-path replication share grows with network scale (4.1% at 50 N to 17.5% at 200 N).

Under 50% fault conditions, FedCARE demonstrates a statistically significant and anticipated increase in energy consumption relative to the baseline methods: 6.82 mJ/pkt versus 6.32 mJ/pkt for RPL-RP at 200 nodes ($p_{\mathrm{holm}}\leq 0.05$, Table 6). This 7.8% increase represents the deliberate cost of resilience. Standard protocols achieve lower energy per packet by dropping packets when disconnected paths are encountered. This inflates their apparent efficiency. In contrast, FedCARE’s confidence-gated resolver and $k_{\mathrm{rep}}$-path replication mechanism instead successfully delivers 1.8× more mission-critical packets across longer alternative paths. This justifies the marginal energy overhead in any safety-critical deployment context. Decomposing the per-packet energy at 200 N (Figure 12), path computation accounts for approximately 63.2% of the total, MAC retries for 19.3%, and $k_{\mathrm{rep}}$-path replication for 17.5%. The replication share grows with network scale (4.1% at 50 N to 17.5% at 200 N) because larger topologies expose more node-disjoint paths, enabling the full $k_{\mathrm{rep}}$ = 3 spatial redundancy.

Although latency and energy overhead are statistically detectable at 50 N under nominal conditions (p<0.05, uncorrected for the individual comparison), the absolute differences (≲3 ms latency, ≲0.5 mJ/pkt energy) are below the measurement resolution of any deployed IIoT energy-harvesting module and are operationally negligible. All such comparisons lose statistical significance after Holm–Bonferroni correction ($p_{\mathrm{holm}}>0.05$). The term “statistically significant” is therefore used exclusively for corrected comparisons throughout this paper. Detectability without operational consequence is noted separately.

#### Summary of Key Findings

FedCARE delivers 93–96% of critical packets under 50% fault injection—and overall PDR is 1.8× better than RPL-RP—at a cost of 7.8% higher energy and 17% higher latency versus the Adaptive Baseline. The prioritization benefit, quantified by RRG (3.9–15.0%), increases under fault injection, confirming that adaptive priority differentiation activates precisely when degradation is most severe. In healthy networks, FedCARE introduces no statistically significant overhead after Holm–Bonferroni correction ($p_{\mathrm{holm}}>0.05$). The FL confidence gate operates in conservative deferral mode throughout the simulation experiments; its contribution to cyber-attack classification is validated separately on Edge-IIoTset (99.0% recall, Table 7).

### 6.6. Reliability–Fairness Trade-Off and RRG

FedCARE’s criticality-conditioned forwarding policy prioritizes class-2 traffic over class-0 traffic during severe fault conditions. This behavior is demonstrated in the per-class delivery breakdown in Figure 3, and the aggregate advantage over all baselines is presented in Figure 2. This deliberate load shedding results in a delivery disparity of approximately 20 percentage points between class-0 (73 to 78%) and class-2 (94 to 96%) traffic under fault injection. In contrast, the task-agnostic baseline achieves nearly uniform delivery across classes.

This disparity is a direct consequence of the criticality-conditioned actuation layer. When the fuzzy engine assigns $c^{★}$ = 2, Algorithm 4 allocates $k_{\mathrm{rep}}$ paths and the full retry budget to the critical packet, consuming channel capacity that would otherwise serve routine traffic. In the current cold-start regime, this prioritization is determined entirely by the fuzzy engine. In extended deployments where $\kappa_{m}$ exceeds γ, the confidence-gated resolver can further escalate class assignments through the conservative threat override, thereby amplifying this effect. The RRG metric explicitly quantifies the benefit of this prioritization.

**Figure 13:**
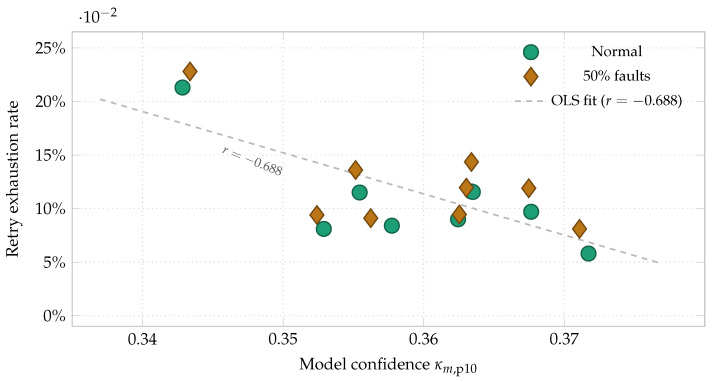
Worst-case inference confidence ($\kappa_{m,p10}$) versus retry exhaustion rate across all 18 FedCARE experimental conditions (r=−0.688). Higher confidence is associated with fewer retry exhaustions in both normal (circles) and 50% fault (diamonds) regimes, confirming that ML inference quality propagates to MAC-layer forwarding behavior.

RRG increases as network stress intensifies, reaching a peak of 15.0% at the 50-node scale under severe fault injection. Under normal conditions, RRG is 3.9% at 100 nodes and 5.7% at 200 nodes. These results confirm that the fuzzy logic controller applies stricter priority differentiation precisely when network degradation is most severe. This contrasts with uniform prioritization regardless of operating conditions, which is a key property of the PCA loop’s adaptive design. A direct relationship exists between worst-case inference confidence and MAC-layer retry behavior across all 18 experimental conditions (r=−0.688), as quantified in Figure 13. As $\kappa_{m,p10}$ increases, the retry exhaustion rate consistently decreases across both fault regimes, confirming that the quality of ML inference propagates to physical forwarding behavior, even in the conservative deferral mode observed in this study.

### 6.7. Bridging the Classification-to-Delivery Gap

While existing IDS designs focus predominantly on classification accuracy, Table 10 exposes a critical gap in how ML models for IIoT security are evaluated: high detection accuracy does not guarantee that the detected alert is actually delivered. Ferrag et al. [24] achieve 95.89% classification accuracy on Edge-IIoTset, but this metric provides no information about whether a correctly classified alarm packet survives a degraded network to reach its destination. FedCARE explicitly closes this gap by coupling its federated inference layer to a criticality-conditioned routing actuation layer, maintaining 92–96% critical delivery across both physical environmental hazards and cyber-attack scenarios. This result directly validates the paper’s central architectural claim: that the reasoning-to-action chain, from fuzzy-supervised federated inference through confidence-gated resolution to deterministic $k_{\mathrm{rep}}$-path forwarding, is sufficient to deliver ≥92% of critical traffic across both hazard types under both cold-start and converged FL conditions, with each stage—fuzzy labeling, confidence gating, and federated escalation—contributing measurably to the result (Table 3; Section 6.9).

### 6.8. Supplementary Analyses: Energy Decomposition, Threshold Sensitivity, and Effect Sizes

**Figure 14:**
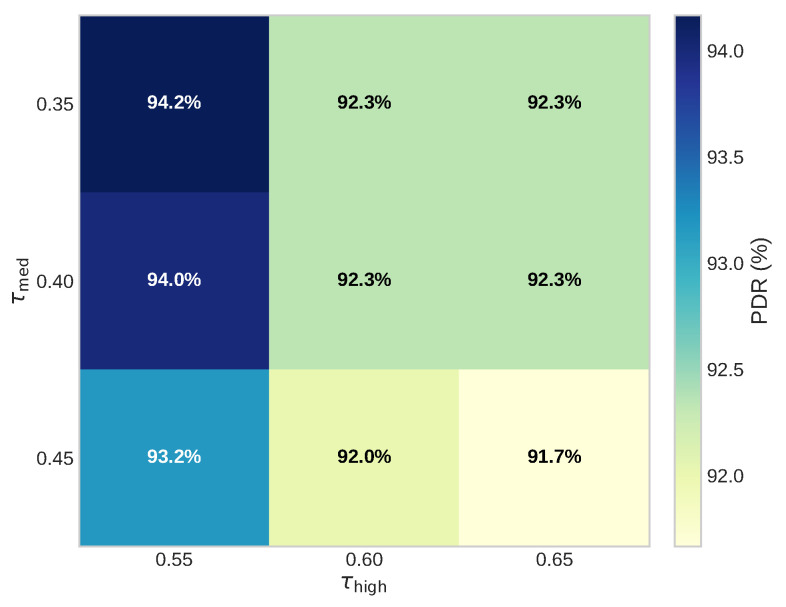
Parameter sensitivity heatmap: overall PDR versus $\tau_{\mathrm{med}}\times \tau_{\mathrm{high}}$ at 100 N under normal conditions with γ = 0.55. PDR varies by only ±2.5 pp across all 18 configurations.

**Figure 15:**
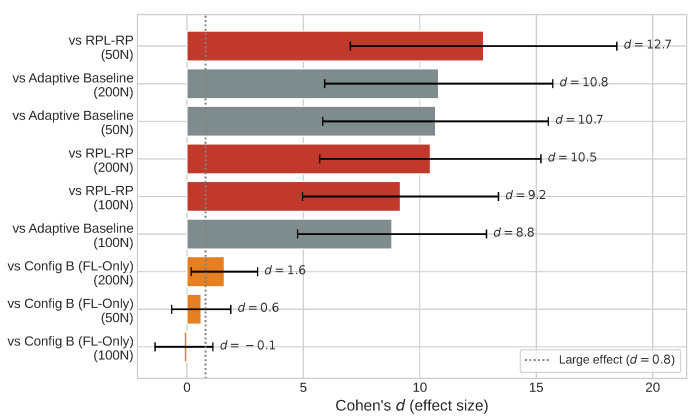
Cohen’s *d* effect sizes with 95% CIs for FedCARE versus each baseline under 50% fault injection.

Three supplementary analyses complement the scalability and resilience results of Section 6.3, Section 6.4 and Section 6.5. Figure 12 decomposes the per-packet energy overhead by component; Figure 14 visualizes the threshold sweep quantified in Section 6.4; and Figure 15 reports the effect sizes underlying the statistical comparisons. The effect-size analysis clarifies the division of labor within the PCA loop: the shared fuzzy engine drives most of the overall-PDR advantage under cold start—FedCARE and Config B are statistically indistinguishable (d≈−0.1 at 100 N)—whereas the large effects against RPL-RP and the Adaptive Baseline (*d* = 8.8–12.7) reflect the criticality-aware prioritization that distinguishes FedCARE from the static protocols.

### 6.9. End-to-End Loop Validation

The cold-start evaluation (Section 6.3, Section 6.4 and Section 6.5) validates the fuzzy engine and $k_{\mathrm{rep}}$-path actuation under conditions where $\kappa_{m,p90}\leq 0.37<\gamma$ = 0.55, so the confidence gate never fires and the FL model contributes no routing decisions. To verify that the full PCA loop is functional when the FL model reaches decisional maturity, we conduct a supplementary experiment in which the MLP is pre-trained to convergence on Edge-IIoTset before the routing simulation begins. Pre-training yields classification accuracy of 69–91% and mean softmax confidence of 0.63–0.88 across seeds and scales, well above the gate threshold. The same topologies, fault regimes, traffic patterns, and five random seeds from the main evaluation are reused; only the model initialization differs.

#### 6.9.1. Gate Activation

Table 11 reports the gate behavior under 50% fault injection. With the converged model, mean routing-phase confidence reaches ${\bar{\kappa }}_{m}$ = 0.71–0.80 ($\kappa_{m,p90}$ = 0.82–0.95), and the confidence gate fires for 88–92% of packets, confirming that the FL model actively participates in routing decisions. Of the gate-fired packets, 8–16% are escalated (i.e., $c_{m}>c_{f}$), and 90% of those escalated packets are successfully delivered. The gate fire rate increases with network scale because more FL clients yield a better-calibrated global model, producing higher per-inference confidence.

#### 6.9.2. Overall and Critical Delivery

Table 12 compares cold-start and converged FedCARE under 50% faults. Overall PDR improves by 1.6–3.5 pp at every network scale, confirming that the federated inference path adds incremental reliability beyond the fuzzy-only baseline. The per-class critical delivery rate decreases slightly (−0.4 to −1.8 pp) because the converged model escalates more packets into the critical class via the monotonic-escalation rule $c^{★}=\max\left(c_{f},c_{m}\right)$: at 200 N, 1167 packets receive critical-class treatment versus 967 under cold start, an increase of 21%. Despite the lower per-class rate, the absolute number of critical packets successfully delivered increases by 20–28% across all scales—the system casts a wider safety net at a modest dilution of per-class delivery. Energy per packet remains effectively unchanged (<0.4% difference), because the converged model replaces fuzzy-only decisions with ML-informed ones rather than adding redundant transmissions.

#### 6.9.3. Interpretation

These results confirm three properties of the PCA loop. First, the confidence gate transitions from complete deferral (cold start, $\kappa_{m,p90}\leq 0.37$) to active participation (converged, gate fire rate 88–92%) without manual reconfiguration, validating the threshold-based design. Second, overall packet delivery improves at every evaluated scale when the FL model contributes, demonstrating that the federated inference path is functional and beneficial rather than dormant. Third, the monotonic-escalation rule $c^{★}=\max\left(c_{f},c_{m}\right)$ operates as intended: the converged model can only promote traffic to a higher criticality class, never demote it, so the safety floor established by the fuzzy engine is preserved. The slight per-class critical PDR dilution (0.4–1.8 pp) is the expected cost of this wider safety margin and is more than offset by the 20–28% increase in absolute critical-packet delivery.

## 7. Limitations

FedCARE demonstrates strong reliability across all evaluated conditions for critical flows. However, seven limitations affect the generalizability of these results and should be clearly disclosed.

First, simulation fidelity is limited. The Bernoulli link-drop model and Watts–Strogatz topology are analytical approximations. Results on IEEE 802.15.4 hardware may differ, as the simulator does not capture factors such as CSMA/CA contention, co-channel interference, and duty-cycling effects.

Second, cold-start federated learning is challenging. Model confidence $\kappa_{m}$ remained below γ = 0.55 in all main experiments (observed $\kappa_{m,p90}\leq 0.37$, a gap of ≥0.18 to the gate threshold). As a result, the FL layer operated in conservative deferral mode, and the routing gains reported in Section 6 are due to the fuzzy engine and $k_{\mathrm{rep}}$-path actuation. Section 6.9 demonstrates that the confidence gate activates for 88–92% of packets when the model is pre-converged, improving overall PDR by 1.6–3.5 pp; however, that experiment assumes offline pre-training, which may not be available in all deployment scenarios. This cold-start property is an intended safety feature of the PCA loop, ensuring robust routing performance before the FL model reaches decision maturity; the fully converged model’s 99.0% critical recall on Edge-IIoTset confirms that the FL layer performs as designed when operating on real attack signatures.

Third, while the seed count has been increased to *n* = 5 seeds, further increasing to *n* ≥ 10 would narrow confidence intervals, particularly at 50 N where stochastic variation in topology generation is most pronounced.

Fourth, using a single topology model limits generalizability. Results are specific to the Watts–Strogatz model (*k* = 6); other topologies common in IIoT deployments, including grid, tree, and random geometric graphs, may exhibit different scaling and path-diversity behavior.

Fifth, the use of synthetic FL training data is a limitation. Client data were synthesized from path metrics. Performance on real, non-IID sensor telemetry was validated for classification on Edge-IIoTset, but not for the full in-network routing loop.

Sixth, there is a throughput trade-off. FedCARE’s throughput is 17–42% lower than the Adaptive Baseline (17% at 50 N, 42% at 100 N, 38% at 200 N) because $k_{\mathrm{rep}}$-path replication consumes channel capacity. This is unfavorable for throughput-saturated applications such as continuous video surveillance. Additionally, per-class delivery analysis reveals a deliberate disparity: class-0 (Low) packets achieve 73–78% delivery, while class-2 (Critical) packets achieve 94–96% under fault injection. This load shedding at the routing layer highlights the need for dynamic fairness tuning during prolonged fault conditions.

Seventh, on-device deployment introduces a quantifiable resource overhead. The Mamdani FIS, configured with 27 rules and trapezoidal membership functions, requires approximately 1.2 KB ROM and 256 B RAM. In comparison, the two-hidden-layer multilayer perceptron (4→64→64→3, 4675 parameters) requires approximately 18.3 KB ROM and 2.5 KB RAM at float32 precision. Inference latency is less than 2 ms for the fuzzy system and approximately 15 ms for the MLP. Since the MLP operates asynchronously between routing epochs rather than inline with each packet, the 15 ms latency does not contribute to per-packet forwarding delay. Each federated round transmits the complete model weights, amounting to approximately 18.3 KB per client. Collectively, these factors result in a 7.8% increase in energy consumption per delivered packet (6.82 vs. 6.32 mJ at 200 N under 50% faults). Under the experimental traffic profile, where routing-related transmissions dominate the energy budget, this corresponds to an estimated 7% reduction in the Zolertia Z1 operational lifetime. These values are calculated analytically based on model parameter counts and the per-transmission energy model described in Section 6.5. Empirical validation on Contiki-NG hardware is planned for future work.

## 8. Conclusions

FedCARE addresses three key machine learning challenges in safety-critical IIoT systems—the absence of interpretable supervision signals, the inability to act conservatively on per-inference confidence, and the brittleness of federated aggregation under partial availability—through three coordinated contributions: a 27-rule Mamdani Fuzzy Inference System that generates domain-consistent criticality labels from multi-modal sensor telemetry via trapezoidal membership functions and centroid defuzzification; a dropout-aware aggregation protocol that normalizes exclusively over the active client set and degrades gracefully under disconnection; and a confidence-gated resolver that applies an auditable maximization rule $c^{★}=\max\left(c_{f},c_{m}\right)$ to prevent under-prioritization of safety-critical traffic. Evaluation across 50-, 100-, and 200-node Watts–Strogatz topologies under fault rates up to 50% demonstrates 99.00% critical recall on the Edge-IIoTset benchmark and up to 1.8× higher high-criticality delivery than RPL-RP under severe 50% node-failure conditions, with no measurable overhead under nominal conditions after Holm–Bonferroni correction.

These results establish fuzzy-supervised federated inference with confidence gating as a principled and effective design paradigm for machine learning in constrained, safety-critical cyber-physical systems. The deliberate architectural choice of node-disjoint $k_{\mathrm{rep}}$-path replication for critical traffic, combined with the conservative deferral behavior of the confidence gate during cold start, ensures that routing gains are attributable to interpretable, auditable mechanisms rather than to opaque learned policies. A supplementary converged-model experiment (Section 6.9) confirms that the confidence gate activates for 88–92% of packets when the FL model reaches decisional maturity, improving overall delivery by 1.6–3.5 pp while increasing the absolute number of critical packets successfully delivered by 20–28%, thereby validating the full PCA loop end-to-end. The observed 7.8% energy overhead and 20 pp inter-class delivery disparity bound the deployment regimes where criticality prioritization is cost-justified, and the seven limitations identified in Section 7 define the scope within which these conclusions hold.

### Future Work

Future research will address the limitations identified in Section 7 by moving from Watts–Strogatz simulation to closed-loop hardware deployment with Zolertia RE-Mote nodes running Contiki-NG. This approach will enable empirical validation of energy models and allow quantification of battery overhead associated with federated synchronization. Generating real cyber-attack traffic during hardware trials will extend the converged-model validation of Section 6.9 to genuine distributional shift, thereby confirming the FL layer’s routing contribution under operational conditions rather than synthetic pre-training. Additionally, future research will investigate adaptive mechanisms for the confidence-gating threshold γ to respond to real-time distributional shifts, and will examine fairness-aware scheduling strategies to limit the delivery gap between criticality classes during extended fault conditions. To reduce the energy overhead of criticality-aware forwarding, future work will explore adaptive $k_{\mathrm{rep}}$ selection, such as reducing the replication factor when $E_{\mathrm{res}}<0.50$, probabilistic path selection to avoid full $k_{\mathrm{rep}}$-path flooding, integration of duty-cycling to amortize idle listening costs, and federated energy-aware load balancing across heterogeneous mote populations. Expanding evaluation to include alternative topology models, such as grid, tree, and random geometric graph, and increasing the seed count to *n* ≥ 10 are immediate priorities to strengthen the statistical basis of the reported results.

## Figures and Tables

**Table 1:** Comparison of FedCARE against related work across the three contribution axes: interpretable inference, federated learning, and learning-augmented routing.

Category & References	Technique & Scope	Critical Limitation	FedCARE Mechanism
Axis I: Interpretable Inference and Knowledge Extraction			
Mixed-Criticality Systems [26]	Static safety levels assigned at design time.	No Adaptivity: Cannot respond to dynamic hazards or distributional shift at runtime.	Dynamic Labeling: Fuzzy supervision generates adaptive criticality labels from live sensor telemetry.
Fuzzy Logic Systems [17,18]	Mamdani inference for control or task allocation.	Standalone Decisioning: Interpretable but not used to supervise or label data for downstream learning.	Knowledge Extractor: FIS labels supervise the federated model.
Explainable CPS [11,12]	DL/XAI for attack attribution.	Passive Diagnosis: Interpretable without closing the reasoning-to-action chain.	Actuation-Coupled: Inferred criticality drives forwarding policy.
Axis II: Federated Learning for Security			
FL-IDS Frameworks [8]	Collaborative training without raw data sharing across sensor nodes.	Accuracy-Centric: No confidence quantification; no coupling to network decisions.	Confidence-Gated FL: Per-inference confidence governs resolver.
Federated Risk Assessment [7,9]	Federated XGBoost/SVM for risk scoring.	Open-Loop: Risk scores not coupled to routing or redundancy; no graceful degradation.	Closed-Loop Control: Risk scores directly modify routing metrics.
Adaptive FL Aggregation [27]	Shapley-value weighting and client selection for non-IID data.	Accuracy-Only: Improves model quality, but does not address cold-start deferral or actuation.	Dropout-Aware Protocol: Normalizes over active-client set; degrades gracefully.
Explainable FL-IDS [10]	FL augmented with SHAP/LIME.	Informational Only: Explanations do not trigger differentiated reliability or routing.	Semantic Actuation: Criticality estimates map to *k*-path replication.
Trustworthy FL for IoT [28]	Digital-twin + blockchain purification of poisoned FL updates.	Integrity-Terminal: Pipeline ends at a clean global model; inference never actuates the network.	Inference-to-Action: Resolved class drives forwarding policy.
Axis III: Learning-Augmented Routing			
ML-Augmented RPL [29,30,31]	RF, Gradient Boosting, Q-learning for parent selection.	Semantic Blindness: Optimizes network metrics without payload urgency awareness.	Semantic Routing: Policy conditioned on inferred criticality class.
Energy-Aware Fault-Tolerant Routing [32]	QoS-aware routing with node fault prediction for IoT sensor networks.	No Threat Intelligence: Adapts to physical node failures but not cyber-physical attack patterns.	Cyber-Physical Co-Design: Redundancy scaled to joint cyber-physical criticality class.
Deep RL Routing [33]	DRL agents optimizing throughput and energy.	Black-Box Decisions: No interpretability; poor convergence under rapid topology change.	Interpretable FIS: Deterministic rule-traceable safety bounding.
Federated DRL Routing [34,35]	FL-coordinated DDQL/federated RL for adaptive path selection in WSN and 5G-IIoT.	Opaque & Class-Blind: Learned policy is non-traceable, provides no per-inference confidence, and treats all packets as equally urgent.	Gated Semantic Policy: Interpretable labels + confidence gate condition forwarding on criticality class.
Intelligent Fault-Tolerant Routing [36]	Hybrid RL detection and recovery of node/link faults in WSN-assisted IIoT.	Element-Level Criticality: Resilience attaches to faulty nodes and links, not to inferred payload urgency; no federated training.	Payload-Level Criticality: $c^{★}$ inferred from telemetry governs replication and retries.
Disaster-Aware Networking [1,37]	Protocols recovering connectivity after physical infrastructure damage.	Unidimensional Resilience: Handles physical node loss but integrates no learned threat intelligence.	Cyber-Physical Co-Design: Redundancy scaled to joint cyber-physical criticality.
FedCARE (Proposed)	Unified closed-loop ML pipeline: fuzzy labeling, dropout-aware FL, confidence gating, semantic actuation.	Not Applicable	Full-Stack Integration: FIS + FL + Confidence Gating + IPv6 Routing.

**Table 2:** Analysis of research gaps, impacts, and constraints in IIoT disaster scenarios.

Research Theme	Specific Research Gaps	Impact on Disaster Scenarios	Evidence-Based Constraints	Source
Data Privacy	Absence of uniform privacy controls across heterogeneous, multi-vendor IIoT sensor platforms. Vulnerability to gradient and parameter leakage during cross-layer transmissions. Lack of robust protection against inference attacks on non-IID industrial data streams.	Leakage of sensitive operational data compromises trust and prevents multi-agency collaboration Heavyweight privacy mechanisms disrupt time-critical control loops.	Strict trade-off between cryptographic overhead, differential privacy noise, and real-time accuracy. High CPU overhead exceeds the capacity of constrained edge sensor nodes.	[7,13,14]
Latency	High synchronization overhead in FL protocols. Straggler effects in synchronous updates. Lack of adaptive aggregation timing for non-stationary sensor environments.	Prevents prompt response in safety-critical actuators. Delays autonomous decisions such as victim localization and hazard mapping.	Severe communication delays due to signal blockages and multipath fading. Bandwidth limits and intermittent connectivity in damaged infrastructure.	[15]
Heterogeneity	Massively non-IID data distributions across sensor node types. Lack of standardized protocols across multi-vendor OT systems. Absence of unified cross-layer SLA frameworks.	Domain shifts cause biased or inaccurate hazard prediction models. Interoperability failures between diverse rescue sensors prevent seamless data fusion.	Severe resource disparities cause convergence instability. Incompatible communication protocols hinder real-time collaboration.	[38]
Standardization	Absence of unified protocols for data exchange between sensor node manufacturers. Deep hardware and software coupling in IIoT edge devices.	Protocol incompatibility stalls rapid model deployment across rescue teams. Hinders trustworthy data flow across organizational boundaries.	Communication mismatches between heterogeneous edge platforms. Lack of common APIs for cross-domain interoperability.	[39,40]

**Table 3:** Component ablation results (200 N, 50% faults, *n*= 5 seeds).

Configuration	Overall PDR	Critical PDR	Energy (mJ/pkt)
Config C: Full FedCARE	**90.4%**	**93.0%**	6.82
Config B: FL-Only ($c^{★}=c_{m}$ always)	84.2%	91.8%	6.54
Adaptive Baseline ($k_{\mathrm{rep}}$ = 1, $r_{\max}$ = 1)	51.1%	—	6.33

**Table 4:** Experimental configuration. All results are reproducible from these parameters using the released simulation code.

Parameter	Symbol	Value
Network and topology		
Network scale (nodes)	—	50, 100, 200
Topology model	—	WS (*k* = 6, *p* = 0.15)
Fault model (link-loss)	$P_{\mathrm{drop}}$	0%, 50%
Random seeds	*n*	5 (seeds 1–5)
Federated learning		
Eligible clients	*K*	N (all nodes)
Active clients/round	—	≈0.85 N (after dropout)
Packets per scenario	—	200 (50/100 N), 400 (200 N)
FL rounds	*R*	4 (50/100 N), 8 (200 N)
Learning rate	η	0.12
Local epochs	*E*	2
Client dropout rate	—	0.15
FL round period	—	50 pkts
Edge-IIoTset critical mix	—	20%
WUSTL-IIoT-2021 attack mix	—	7%
Fuzzy inference and confidence gating		
Medium/high threshold	$\tau_{\mathrm{med}}$/$\tau_{\mathrm{high}}$	0.40/0.60
Confidence gate	γ	0.55
Adaptive routing		
Replication/max retries	$k_{\mathrm{rep}}$/$r_{\max}$	3/3
Timeout/burst loss prob.	$P_{\mathrm{timeout}}$/$P_{\mathrm{burst}}$	0.03/0.50
Burst period/length	—	400/25 pkts
Min. energy for replication	$E_{\min}$	0.20

**Table 5:** Notation used in the edge-intelligence pipeline.

Symbol	Description
S	Local sensor data stream.
C	Routing protocol configuration parameters.
*p*	Data packet generated by a source node.
γ	Confidence threshold for accepting the ML model’s criticality prediction.
src,dst	Source node and sink destination for *p*.
$G_{\mathrm{local}}=\left(V,E\right)$	Local communication graph with weighted links.
λ,ε,δ	Path metrics: latency-related, energy-related, and distance-related costs, respectively.
M	Membership-function set used in fuzzification.
R	Mamdani fuzzy rule base.
$T=\{\tau_{\mathrm{med}},\tau_{\mathrm{high}}\}$	Discretization thresholds for medium- and high-criticality classes.
$\sigma_{f},c_{f}$	Fuzzy urgency score and corresponding class output.
F	Packet-level feature vector for model inference.
$\Theta^{\left(r\right)}$	Global federated model at aggregation round *r*.
$\theta_{k}^{\left(r\right)}$	Local model weights for client *k* at round *r*.
$K,K_{r}$	Total number of clients and the active-client subset participating in round *r*.
E,η	Number of local training epochs and learning rate.
$\sigma_{m},c_{m},\kappa_{m}$	Model urgency score, model class, and model confidence.
$c^{★}$	Final resolved criticality class used by the routing layer.
$E_{\mathrm{res}}$	Residual battery level at the source node.
$E_{\min}$	Minimum residual energy required to enable high-criticality path replication.
$r_{\max}$	Maximum retry budget for medium- and high-criticality packets.
$k_{\mathrm{rep}}$	Replication factor: number of candidate paths computed for high-criticality forwarding.
P,π	Candidate path set and an individual path within that set.
Ack	Transmission acknowledgment flag.
$N_{\mathrm{attempts}}$	Number of transmission attempts assigned by the routing policy.
$P_{\mathrm{timeout}},P_{\mathrm{burst}}$	Baseline drop probability under normal conditions and elevated drop probability during a burst-failure window, respectively.
$\ell_{r},{\mathrm{Acc}}_{r}$	Validation loss and accuracy recorded at FL round *r*.

**Table 6:** Performance comparison of state-of-the-art protocols versus FedCARE under severe fault conditions (50% node loss).

Protocol	Overall PDR (%)			Critical PDR (%)			Energy (mJ/pkt)			Latency (ms)		
	50 N	100 N	200 N	50 N	100 N	200 N	50 N	100 N	200 N	50 N	100 N	200 N
RPL [20]	35.1	31.0	10.4	50.7	42.0	12.8	21.5	23.5	29.9	58.0	72.8	72.2
LOADng [51]	35.1	31.0	13.7	50.7	42.0	12.8	21.5	23.5	31.4	57.9	72.8	82.1
EL-RPL [52]	35.6	31.5	12.0	50.7	42.0	12.8	21.6	23.5	28.6	58.2	72.8	79.8
RPL-RP [21]	58.3	50.7	51.1	—	—	—	4.8	6.2	6.3	48.1	53.3	63.9
DRL-Routing [33]	38.5	35.0	15.1	43.0	36.5	15.1	26.8	31.2	38.5	68.4	84.5	91.2
FedCARE	87.7	87.6	90.4	95.9	93.3	93.0	5.3	6.8	6.8	51.0	55.7	66.1

**Table 7:** FedCARE detection performance on Edge-IIoTset.

Metric	Value	Interpretation
Accuracy	99.00%	High overall classification correctness.
Precision	0.9901	Low false-positive rate.
Recall	0.9900	Near-zero missed critical alarms.
F1-Score	0.9899	Balanced precision–recall performance.

**Table 8:** Effect sizes (Cohen’s *d*) for FedCARE vs. Adaptive Baseline. Values *d* > 0.8 are large; *d* > 2 are very large [53]. The extreme Critical PDR values at 200 N reflect near-zero variance across seeds, not measurement error.

Metric	Regime	50 N	100 N	200 N
PDR	Normal	3.22	6.61	11.92
PDR	Faults50	3.72	8.03	11.41
Critical PDR	Normal	8.53	10.93	46.19
Critical PDR	Faults50	8.61	14.68	37.50
Latency	Normal	0.53	0.84	0.30
Energy	Normal	0.30	1.28	0.61
Burst SR	Faults50	2.75	7.79	4.16

**Table 9:** Overall PDR (%) sensitivity to fuzzy thresholds at 100 N (mean over *n* = 3 seeds; γ omitted as it has no effect under cold-start).

$\tau_{\mathrm{med}}$	$\tau_{\mathrm{high}}$		
	0.55	0.60	0.65
0.35	94.2	92.3	92.3
0.40	94.0	**92.3**	92.3
0.45	93.2	92.0	91.7

**Table 10:** IDS accuracy benchmarks versus FedCARE critical delivery reliability, illustrating the classification-to-delivery gap.

Dataset	Study/Model	Metric	Value (%)
*Edge-IIoTset*			
Ferrag et al. [24]	FL (15-class)	Accuracy	95.89
FedCARE	Criticality-aware Routing	Critical PDR at 30% loss	94.31
*WUSTL-IIoT-2021*			
Zolanvari et al. [54]	Centralized ANN	Accuracy	99.98
FedCARE	Criticality-aware Routing	Critical PDR (DoS)	93.99

**Table 11:** Confidence-gate behavior with a pre-converged FL model under 50% fault injection (mean ± std over *n* = 5 seeds).

Metric	50 N	100 N	200 N
Model confidence ${\bar{\kappa }}_{m}$	0.71	0.75	0.80
Confidence $\kappa_{m,p90}$	0.82	0.91	0.95
Gate fire rate (%)	88.4	90.1	92.4
Escalation rate (%)	8.0	16.3	16.3
Escalation delivery rate (%)	91.4	90.5	90.4

**Table 12:** Cold-start versus converged FedCARE under 50% fault injection (mean over *n* = 5 seeds). Δ pp denotes the percentage-point difference.

		50 N	100 N	200 N
Overall PDR (%)	Cold-start	87.7	87.6	90.4
	Converged	90.2	91.1	92.0
	Δ	+2.5	+3.5	+1.6
Critical PDR (%)	Cold-start	95.9	93.3	93.0
	Converged	95.5	91.4	92.2
	Δ	−0.4	−1.8	−0.8
Critical pkts delivered	Cold-start	98	292	899
	Converged	118	375	1076
	Abs. gain	+20%	+28%	+20%
Energy (mJ/pkt)	Cold-start	5.28	6.79	6.82
	Converged	5.32	6.60	6.84

## Data Availability

The simulation pipeline, configuration files, and seed-level raw results supporting this study will be made available at a public repository upon acceptance. All reported values are reproducible from a single invocation of the simulation pipeline described in Section 5.

## Abbreviations

FedCARE	Federated Criticality-Aware Routing Engine
IIoT	Industrial Internet of Things
FL	Federated Learning
RPL	Routing Protocol for Low-Power and Lossy Networks
ML	Machine Learning
IPv6	Internet Protocol version 6
FIS	Fuzzy Inference System
SHAP	SHapley Additive exPlanations
LIME	Local Interpretable Model-agnostic Explanations
IDS	Intrusion Detection System
FL-IDS	Federated Learning Intrusion Detection System
XAI	Explainable Artificial Intelligence
LLN	Low-Power and Lossy Network
MAC	Medium Access Control
RSSI	Received Signal Strength Indicator
CPS	Cyber-Physical Systems
RL	Reinforcement Learning
DRL	Deep Reinforcement Learning
FedAvg	Federated Averaging
non-IID	Non-Independent and Identically Distributed
SDN	Software-Defined Networking
DoS	Denial of Service
DDoS	Distributed Denial of Service
QoS	Quality of Service
PCA	Perception–Cognition–Actuation
MLP	Multilayer Perceptron
UDGM	Unit Disk Graph Medium
CSMA/CA	Carrier Sense Multiple Access with Collision Avoidance
KPI	Key Performance Indicator
PDR	Packet Delivery Ratio
RRG	Relative Reliability Gain
SR	Success Rate
EDP	Energy-Delay Product
OLS	Ordinary Least Squares
CI	Confidence Interval
